# Interpretable Deep-Learning p*K*_a_ Prediction for Small Molecule Drugs via Atomic Sensitivity Analysis

**DOI:** 10.1021/acs.jcim.4c01472

**Published:** 2024-12-30

**Authors:** Joseph DeCorte, Benjamin Brown, Rathmell Jeffrey, Jens Meiler

**Affiliations:** † Department of Chemical and Physical Biology, Vanderbilt University, Nashville, Tennessee 37232, United States; ‡ Center for Structural Biology, Vanderbilt University, Nashville, Tennessee 37232, United States; § Vanderbilt Medical Scientist Training Program, Vanderbilt University Medical Center, Vanderbilt University School of Medicine, Nashville, Tennessee 37232-8725, United States; ∥ Department of Chemistry, Vanderbilt University, Nashville, Tennessee 37232-8275, United States; ⊥ Center for Applied AI in Protein Dynamics, Vanderbilt University, Nashville, Tennessee 37232-8725, United States; # Department of Pathology, Microbiology, and Immunology, Vanderbilt University Medical Center, Nashville, Tennessee 37232, United States; ∇ Institute for Drug Discovery, Leipzig University Medical School, Leipzig, SAC 04103, Germany

## Abstract

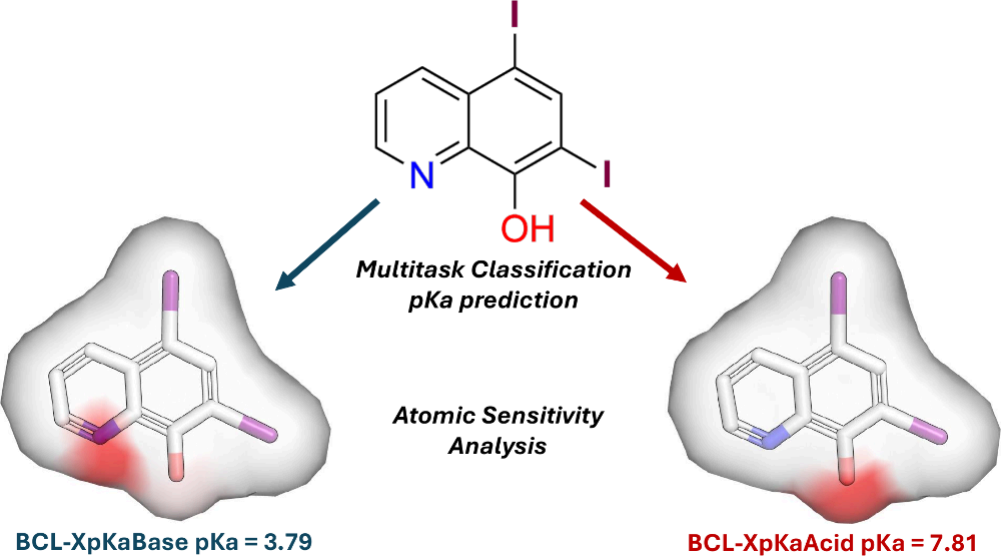

Machine learning (ML) models now play a crucial role in predicting properties essential to drug development, such as a drug’s logscale acid-dissociation constant (p*K*_a_). Despite recent architectural advances, these models often generalize poorly to novel compounds due to a scarcity of ground-truth data. Further, these models lack interpretability. To this end, with deliberate molecular embeddings, atomic-resolution information is accessible in chemical structures by observing the model response to atomic perturbations of an input molecule. Here, we present BCL-XpKa, a deep neural network (DNN)-based multitask classifier for p*K*_a_ prediction that encodes local atomic environments through Mol2D descriptors. BCL-XpKa outputs a discrete distribution for each molecule, which stores the p*K*_a_ prediction and the model’s uncertainty for that molecule. BCL-XpKa generalizes well to novel small molecules. BCL-XpKa performs competitively with modern ML p*K*_a_ predictors, outperforms several models in generalization tasks, and accurately models the effects of common molecular modifications on a molecule’s ionizability. We then leverage BCL-XpKa’s granular descriptor set and distribution-centered output through atomic sensitivity analysis (ASA), which decomposes a molecule’s predicted p*K*_a_ value into its respective atomic contributions without model retraining. ASA reveals that BCL-XpKa has implicitly learned high-resolution information about molecular substructures. We further demonstrate ASA’s utility in structure preparation for protein–ligand docking by identifying ionization sites in 93.2% and 87.8% of complex small molecule acids and bases. We then applied ASA with BCL-XpKa to identify and optimize the physicochemical liabilities of a recently published KRAS-degrading PROTAC.

## Introduction

Predicting a drug’s behavior in the body is a key challenge in computational drug development. For example, accurate prediction of a compound’s bioavailability could support early modification or termination of nonviable lead molecules, thereby saving years of time and millions of dollars on research and development. The demand for fast and accurate predictions of a drug’s quantitative structure–activity and structure–property relationships (QSAR, QSPR) has skyrocketed as our access to synthesizable chemical space approaches one trillion molecules.^1^ While advances in machine learning (ML) have improved prediction accuracy, the small amount of publicly available, high-quality experimental data for training often leads to overfitting and prevents generalizability.^2−4^ Further, QSPR model interpretability is often poor, despite the relatively more intuitive input (chemical structures) than in other fields of computational biology (e.g., transcriptomics data). As such, additional explorations into architectures that can efficiently train on chemical data, as well as general methods to interpret these models’ outputs, are warranted.

One of the most critical properties to a drug’s downstream efficacy is its ionizability at physiologic pH values, which depends on the drug’s logscale acid-dissociation constant (p*K*_a_ value).^4,5^ Quantum mechanical (QM) methods now calculate p*K*_a_ with experimental accuracy and are extremely valuable to late-stage drug development. However, small-molecule drug development often begins with virtual high-throughput screening (vHTS) of billions of compounds, and QM methods are too computationally expensive to assist meaningfully in vHTS. As such, scientists have made tremendous investment in ML-based QSAR/QSPR predictors for faster, though potentially less accurate, prediction of physicochemical properties like p*K*_a_.

These ML methods generally embed molecules using molecular fingerprints, which are two-dimensional (2D) or 3D chemical substructures centered around each atom in the molecule. Recently, groups have realized significant gains in prediction accuracy with graph neural networks (GNNs), which embed molecules as a graph in addition to standard chemical descriptors.^6−8^ Improvements in molecular featurization strategies (e.g., the use of semiempirical QM-based descriptors^9^), network architecture, and training strategy (e.g., transfer learning on experimental data^9−11^) have driven state-of-the-art p*K*_a_ prediction accuracy to within 0.50–1.00 p*K*_a_ units of experimental values.

Despite these advances, several limitations persist in ML-based p*K*_a_ prediction and QSPR prediction generally. First, all ML-based p*K*_a_ predictors to date use regression. While regression is the natural setting for predicting continuous values, small training set sizes restrict the accuracy of regression outputs, particularly at extreme, but still physically relevant, values.

Second, the model generalizability remains poor. To date, we have only been able to collect experimental data for ∼10^3^ small molecules, which is too limited to robustly train a p*K*_a_ predictor. As such, nearly all p*K*_a_ predictors use large amounts of predicted p*K*_a_ values for small molecule repositories like ChEMBL (∼10^6^ molecules). It is unlikely that we will increase the throughput of the experimental p*K*_a_ value calculation 1000-fold; therefore, strategies of identifying poorly predicted pharmacophores to guide targeted experimental efforts are warranted.

Several attempts at QSPR model interpretability have been explored, but significant challenges remain. Existing methods largely focus on feature-set-level analysis, encompassing stepwise,^12,13^ feature-masking,^14^ feature-set-perturbation,^15^ feature-attribution,^16^ and response-randomization^17^ methods. Feature-set-level analysis is often slow and unintuitive, as feature sets are often large and complex.

More intuitively, several groups have developed approaches using masking or perturbation to interpret the chemical prediction models. Riniker and Landrum developed “similarity maps” that visualize atomic contributions by removing bits associated with each atom from molecular fingerprints and observing changes in similarity or model predictions.^18^ Sheridan later demonstrated that while such atom coloring approaches can provide intuitive visualization, their interpretation is highly sensitive to both descriptor choice and modeling method, requiring very high predictive accuracy to reliably recover known atomic contributions.^19^ Polishchuk et al. also proposed a universal model-agnostic approach that calculates both local (per-molecule) and global (data set-wide) fragment contributions by analyzing prediction differences when fragments are removed.^20^ Indeed, while these methods have greatly advanced our understanding of model interpretability, perturbing an input chemical structure may have unintuitive effects on the resulting descriptors used to featurize molecules.

More recently, Matveieva and Polishchuk showed that atom masking of chemical structure inputs to GNNs can provide atomic-level interpretability, but that GNNs generally have worse interpretability performance than traditional models, in part due to their complex internal molecular representations.^21^ Thus, a method for high-resolution atomic sensitivity analysis is warranted. To this end, chemical structures are privileged data in computational biology in that they can be perturbed in consistent, physical, meaningful ways. With an appropriately constructed feature set, measuring a model’s response to these perturbations would provide granular details into both model learning and molecular hotspots for prediction in real time, without the need for model retraining. For example, replacing a molecule’s acidic carboxylic acid functional group with an inert ketone increases the p*K*_a_ from ∼4 to ∼20, thereby demonstrating the carboxylic acid’s importance to acidity. As a counterexample, increasing or decreasing the expression of a gene in a transcriptomics-based predictor of cellular activity may not be physically meaningful, as gene expression is highly dependent on the network of expressed genes in a cell/tissue. Interestingly, Sushko et al.^200^ leveraged this idea for QSAR model interpretability via “prediction-driven” matched molecular pair (MMP) analysis, which attempts to optimize a property (e.g., toxicity) by perturbing a molecule using a large library of chemical transformations (e.g., changing an ether to a piperazine linkage) and assessing the impact of this perturbation on a model’s output {Sushko, 2014 #52}. However, the authors note that their method poorly accounts for the chemical context of these perturbations. Alternatively, leveraging a similar approach to simply determine the influence of each of a molecule’s atoms or substructures on a desired QSAR property could provide valuable insights into model learning, performance, and molecular structure.

To address these limitations, we present BCL-XpKa, a multitask classifier (MTC) trained with local atomic-environment descriptors for rapid and accurate p*K*_a_ prediction within the Biology and Chemistry Library (BCL), an open-source cheminformatics platform developed and maintained by our lab. We demonstrate that discretizing p*K*_a_ prediction has competitive prediction accuracy without meaningful information loss to state-of-the-art prediction platforms, with the added benefit of directly reading out the model’s uncertainty in its prediction for each molecule. This uncertainty allows us to easily identify regions of the chemical space that are poorly understood by our model. We further show that BCL-XpKa’s atomic-level encodings support superior model generalization to both common molecular fingerprint strategies.

We then leverage BCL-p*K*_a_’s discrete probability distributions and atomic-level encodings with a new method of QSPR model interpretability that we term atomic sensitivity analysis (ASA). We benchmark ASA and demonstrate that it provides atomic-level insights into which regions of a molecule are most important for the model’s final prediction at higher resolution than existing methods for atomic-level interpretability. We also explore ASA’s utility in probing model learning. Finally, we show that ASA can guide the targeted optimization of druglike molecules.

BCL-XpKa is a multilayer perceptron (MLP) that embeds molecules using 2D chemical descriptors features that only encode information about each atom’s local environment (up to 1 bond away) in the molecule.^22^ This scheme enables increased sensitivity to atom-level perturbations, which is particularly important for hit-to-lead and lead optimization in late-stage drug development. Small-molecule drugs often have both basic and acidic regions that vary greatly in p*K*_a_ values (e.g., amino acids have both an acidic carboxylic acid and a basic free amine group). To account for this, we trained two models: one to predict a molecule’s most acidic p*K*_a_ value (BCL-XpKaAcid) and one to predict its most basic p*K*_a_ value (BCL-XpKaBase). We trained BCL-XpKa on data sets of both predicted and experimental p*K*_a_ values, and we evaluate our models on an external test set of challenging acids and bases with experimental p*K*_a_ values. We find that our model is competitive with state-of-the-art p*K*_a_ predictors, including GNN-based models, in terms of accuracy and recapitulating molecular trends in p*K*_a_ values.

Overall, the work presented here has the following contributions to the field:

- We developed a novel framework for QSPR prediction that uses local atomic environment embeddings and replaces regression with multitask classification, using p*K*_a_ prediction to illustrate competitive performance with modern ML models.
- We developed a method that rapidly assesses QSPR model learning and provides atomic-level insights to molecular ionizability without requiring model retraining
- We integrate these two tools in a workflow for lead optimization and apply it to optimize a pan-KRAS degrading Proteolysis Targeting Chimera (PROTAC).

## Results

### BCL-XpKa Model Overview

**Figure 1:**
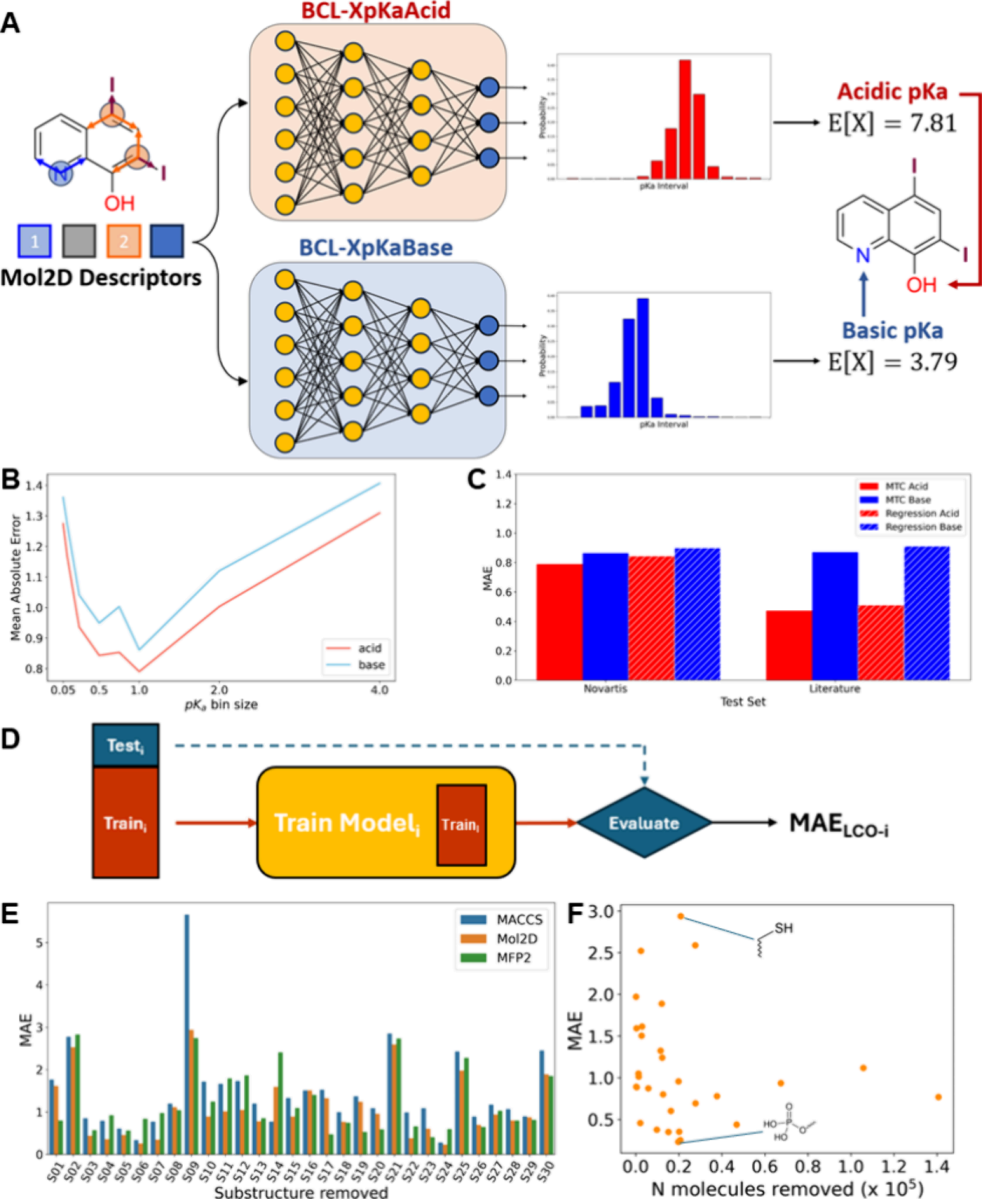
Evaluation of BCL-XpKa’s architecture. (A) BCL-XpKa uses separate models for predicting a molecule’s acidic and basic p*K*_a_ values. It embeds molecules using Mol2D local atomic environment descriptors and then uses an MLP to classify the p*K*_a_ value’s membership in 1-p*K*_a_-unit intervals. Bin edges alternate inclusivity and exclusivity of end points. Extreme bins (p*K*_a_ ≤ 0, p*K*_a_ > 12) are left open on the unbounded side. (B) Multitask classification for p*K*_a_ prediction error with increasing bin size. Small bins allow more precision but fewer data per bin, while larger bins provide lower precision but more data per bin. (C) Performance on two external test sets for BCL-XpKa vs the best-performing regression architecture trained with the same molecule descriptors and training sets. (D) The LCO approach, in which a molecular substructure is removed from model training and used later as a structurally novel test set. (E) Model error was determined by LCO substructure and descriptor type. (F) Model error for an LCO substructure vs the number of TS-Acid or TS-Base molecules containing that substructure.

BCL-XpKa predicts a molecule’s most acidic and most basic p*K*_a_ values using dedicated, MLP-based acid and base models (BCL-XpKaAcid, BCL-XpKaBase). The training sets for BCL-XpKaAcid and BCL-XpKaBase, and TS-Acid and TS-Base, are summarized in Table 1. Briefly, the core of each consists of all molecules in the ChEMBL27 data set with a chembl_acid_pka or chembl_base_pka prediction from ACD/Laboratories, respectively. TS-Acid is augmented with 250 K synthetic acids created to balance the training data, and both TS-Acid and TS-Base are augmented with 60K nonionizable, druglike molecules as negative data (see Methods). This paired model framework allows BCL-XpKa to make unambiguous predictions for both monoprotic compounds and compounds with one acidic and one basic ionization site (Figure 1A). However, BCL-XpKa’s predictions for molecules with multiple acidic or basic ionization centers can be ambiguous. We mitigate some of this ambiguity through atomic sensitivity analysis below, but in general, these molecules are not supported by BCL-XpKa.

**Table 1:** Descriptions of Each Set of Small Molecules Used in BCL-XpKa Training and Evaluation^a^

Set	N mols	Description	Sources
*Training*			
TS-Acid	934,258	Training BCL-XpKaAcid	Ch-Acid + Syn-Acid + Syn-Neg
TS-Base	758,550	Training BCL-XpKaBase	Ch-Base + Syn-Neg
TS-EO-Acid	2776	Acids with experimentally validated p*K*_a_ values	Baltruschat et al.,^23^ Yu et al.,^24^ Liao et al.^25^
TS-EO-Base	3947	Bases with experimentally validated p*K*_a_ values	Baltruschat et al.,^23^ Yu et al.,^24^ Liao et al.^25^
Ch-Acid	645,055	All ChEMBL27 molecules with a chembl acid pka value	ChEMBL
Ch-Base	714,536	All ChEMBL27 molecules with a chembl base pka value	ChEBML
Syn-Acid	257,096	Molecules made in the BCL, consensus predicted p*K*_a_ range 4–8	Made for BCL-XpKa
Syn-Neg	60,000	Molecules made in the BCL with no ionization site	Made for BCL-XpKa
			
*Testing*			
Novartis Acid	108	Several classes of monoprotic and multiprotic acids	Liao et al.^25^
Novartis Baae	168	Several classes of monoprotic and multiprotic bases	Liao et al.^25^
Literature-Acid	26	Generally monoprotic carboxylic acids	Baltruschat et al.^23^
Literature-Base	97	Generally monoprotic amines. Some aromatic heterocycles	Baltruschat et al.^23^
Oxygen-Acids	580	Phenols and carboxylic acids. p*K*_a_ range 0.38–12.23	Yu et al.^24^
Nitrogen-Bases	563	Nitrogen bases. p*K*_a_ range −5.00 to 11.72	Yu et al.^24^
SAMPL6	24	Multiprotic compounds inspired by kinase inhibitors	Işık et al.^26^
SAMPL7	20	Acylsulfonamide compounds and related bioisosteres	Bergazin et al.^27^
SAMPL8	23	Challenging, multiprotic druglike compounds	Bahr et al.^28^

a TS = Test Set. EO = Experimental Only, denoting that all molecules in this set have experimentally determined p*K*_a_ values. Molecules made for Syn-Acid were retained if the deprecated BCL-XpKaAcid model (trained only on Ch-Acid) and ACD/Labs Percepta agreed within 0.5 p*K*_a_ units of one another. The final p*K*_a_ value is the average of these consensus predictions.

BCL-XpKa is largely trained on predicted p*K*_a_ values, given the scarcity of publicly available ground-truth data. Figures S1–S3 contain physicochemical descriptions of these training sets. As an important comparator, we trained the same models solely with small molecules with experimentally determined p*K*_a_ values, referred to as Experimental-Only (EO)-Acid and EO-Base.^23^

Beyond its bespoke training data sets, BCL-XpKa’s key methodological differences from existing models are (1) its multitask classifier architecture and (2) its Mol2D descriptor set, which encodes local atomic environments for each atom in a molecule. Here, we test the effect of these features of model accuracy and generalizability on novel chemical scaffolds.

### Multitask Classification Is Competitive with Regression for Small-Molecule p*K*_a_ Prediction

First, BCL-XpKa reframes p*K*_a_ prediction as a multitask classification (MTC) problem rather than a regression one. Rather than predict p*K*_a_ values on a continuous range, BCL-XpKa predicts the probability that a molecule’s p*K*_a_ lies within discrete intervals, creating a discrete probability distribution of p*K*_a_ values. This distribution’s expected value is BCL-XpKa’s predicted p*K*_a_ value for the molecule (Figure 1A). BCL-XpKa’s MTC approach aggregates the training set into discrete bins, which necessarily leads to some information loss. As bin size decreases, precision increases, but there are fewer points per bin on which to train, so prediction error increases (Figure 1B). Similarly, as bins become larger, there are more data to predict bin membership, but recovering an accurate p*K*_a_ value becomes impossible, so the prediction error again increases (Figure 1B). A bin size of 1 p*K*_a_ unit yielded the lowest MAE on the Novartis-Acid and -Base test sets, which is used in the BCL-XpKa production model.

The benefit of this approach is that it increases the data available at each p*K*_a_ range compared with the equivalent regression task on a continuous interval. For both acids and bases, BCL-XpKa marginally outperforms the best-performing regression models trained on the same data (MAE on Novartis acids: 0.79 vs 0.83; bases: 0.86 vs 0.92, Figure 1C).

Due to its MTC architecture, BCL-XpKa automatically stores its confidence in each prediction as the standard deviation of that molecule’s output p*K*_a_ distribution. This feature directly informs where the model is pathologically confident, which can guide modifications to the training data or model architecture. For example, BCL-XpKaAcid had high confidence (σ ≤ 1.0) and high error (greater than 1.0 p*K*_a_ unit) in 10.3% (n = 11) of the Novartis-Acid set, and BCL-XpKaBase had 10.7% (n = 18) on the Novartis-Base set (Figure S4A-B). Even in this small test set, the pathological Novartis-Acid predictions are enriched for sulfur-containing aromatic heterocycles (5/11, 55.6%). The pathologic Novartis-Base predictions had no such clear trend, likely due to the small size of the test set.

### BCL-XpKa’s Local Atomic Descriptors Facilitate Generalization to Novel Ionizable Substructures

Second, BCL-XpKa uses Mol2D atomic descriptors, a descriptor set created by our lab which counts the occurrences of unique atomic environments (AE) within a molecule, where each AE represents an atom and its neighboring atoms up to 1 bond-length away (Figure 1A, left). Our lab previously demonstrated that Mol2D descriptors are superior to typical fingerprint-based descriptors for several QSAR/QSPR prediction tasks, but p*K*_a_ prediction was not evaluated.^22^ Hence, we retrained BCL-XpKa using radius 2 Morgan Fingerprints (MFP-2) and Molecular ACCess System key (MACCS) embeddings. We retrained and tested all models in a leave-class-out (LCO) fashion, in which all molecules with one of 30 ionizable substructures were withheld from training and these restricted models evaluated on the (now structurally novel) withheld molecules (Figure 1D, E). BCL-XpKa demonstrates robust performance on this LCO test, with an average MAE of 1.1 p*K*_a_ units across all withheld substructures. MACCS and MFP-2 embeddings yield systematically worse results and average MAE values of 1.46 and 1.20 p*K*_a_ units, respectively. No correlation between the size of the withheld set and the resulting model error was observed (Figure 1F). However, BCL-XpKa had the highest error on the −CSH substructure and the lowest error on the phosphate group (−PO_4_). We hypothesize this difference may be attributable to the relative physicochemical homogeneity of phosphorus-containing substructures (e.g., phosphonates) compared to sulfur-containing substructures (e.g., thiazolidinedione rings vs thiophenols).

Importantly, the Mol2D descriptor set also allows BCL-XpKa to store atomic-level insights into molecular ionizability, similar to a GNN, while it uses a simpler MLP architecture. This atomic information is readily extractable, which we demonstrate through an atomic sensitivity analysis (see below).

### BCL-XpKa Is Competitive with State-of-the-Art p*K*_a_ Predictors on a Variety of Tasks

Despite its relatively simple architecture, BCL-XpKa achieves competitive performance to the modern machine-learning p*K*_a_ predictors on external test sets of both acids and bases (Table 2).^7,29−32^ Further, BCL-XpKa outperformed equivalent models trained with a regression architecture (with SAMPL7 as an unexplained and notable exception) and on experimental-only data.

**Table 2:** Head-to-Head Comparison of BCL-XpKa with Other p*K*_a_ Predictors, Equivalent Regression Models, and Equivalent Models Trained Exclusively on Experimental Data^a^

Model	Novartis-Acid	Novartis-Base	Literature-Acid	Literature-Base	SAMPL6	SAMPL7	SAMPL8
BCL-XpKa	0.79	0.8641	0.473	0.869	0.921	0.701	0.864
BCL-MLP-MTC-EO	1.506	1.354	0.966	1.23	1.23	1.34	1.575
BCL-MLP-Regression	0.8434	0.8984	0.5102	0.9104	0.91	0.487	0.862
ChemAxon Marvin	0.808	0.835	0.87	0.87	1.007	0.559	1.3
QupKake	0.79	0.79	0.54	0.54	0.32	0.67	0.62
pKasolver	0.71	0.71	0.52	0.52	–	–	1.244
MolGpKa	0.849	0.789	–	–	0.522	0.797	0.835
Uni-p*K*_a_	0.81	0.493	–	–	0.554	0.57	0.631
MF-SuP-p*K*_a_	0.85	0.61	–	–	0.687	0.656	–
OPERA	1.75	1.71	–	–	0.97	2.135	–
Epik Classical	0.99	0.876	–	–	0.784	1.121	–
ACD/Laboratories Percepta	1.06	0.81	–	–	0.55	–	–
SPOC + XGBoost	–	–	–	–	0.767	1.476	1.108
SPOC + NN	–	–	–	–	0.832	0.932	–
GraphpKa	–	–	–	–	1	0.758	0.916

a Mean absolute error (MAE) displayed where available. For BCL-XpKa, predictions on acids were made with BCL-XpKaAcid and predictions on bases with BCL-XpKaBase. For molecules with multiple acidic or basic ionization centers (e.g., SAMPL6: SM06, SM22), both models were used. BCL-XpKa and BCL-MLP-MTC-EO trained as stated in Table 2. BCL-MLP-Regression trained on TS-Acid and TS-Base, respectively. References for p*K*_a_ values were taken from literature: ChemAxon, QupKake,^9^ pKasolver, MolGpKa,^7^ UnipKa,^10^ MF-SuP-pKa,^33^ OPERA,^32^ Epik Classical,^6^ ACD/Labs Percepta,^29^ SPOC + XGBoost, SPOC + NN, GraphpKa.^8^

### BCL-XpKa Accurately Captures Complex Trends in Ionizability for Druglike Small Molecules

**Figure 2:**
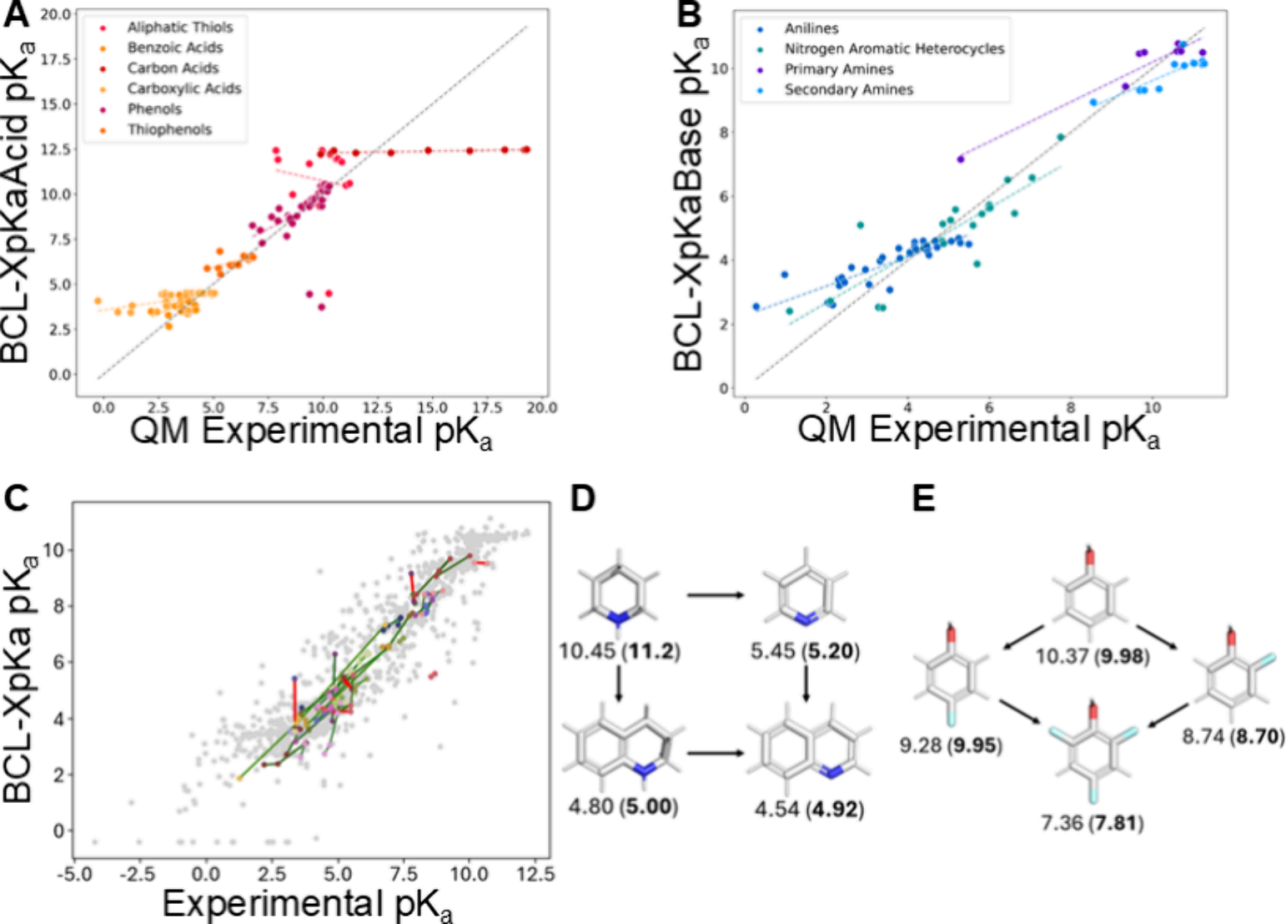
BCL-XpKa external performance and molecular series. (A, B) BCL-XpKaAcid (A) and Base (B) performances on the Thapa molecular series. Trendlines provided for each series, as well as an ideal prediction line. (C) BCL-XpKa prediction vs experimental p*K*_a_ value for families of related druglike molecules in the Oxygen-Acid and Nitrogen-Base sets. These molecules differ by minor chemical modifications. Green denotes that BCL-XpKa correctly predicted the change in p*K*_a_ induced by the chemical modification, and red denotes an incorrectly predicted change in p*K*_a_. (D, E) Example molecular families from (C), with predicted p*K*_a_ shown and experimental p*K*_a_ in parentheses.

While useful, the external test sets used above (Novartis, Literature, SAMPL6–8) are small, introducing potential sampling bias and limiting insights into performance by a specific substructure (excepting SAMPL7). Here, we test BCL-XpKa’s ability to generalize using (1) several series of structurally related molecules and (2) iteratively removing structurally related compounds from training. Recently, Thapa and Raghavachari estimated p*K*_a_ values for several molecular series using a high-level theory QM approach, which the authors of QupKake benchmarked their p*K*_a_ predictor against.^9,34^ QupKake is a GNN that utilizes semiempirical QM features and has superior accuracy to nearly all models on the challenging SAMPL6–8 sets. When evaluating BCL-XpKaAcid and Base on the Thapa sets, we iteratively removed a molecular series from the training set and retrained the full model, similar to the LCO method described above. By comparison, BCL-XpKa outperforms QupKake on aliphatic thiols (1.97 vs 5.09), benzoic acids (0.424 vs 1.45), carboxylic acids (1.01 vs 1.66), phenols (0.61 vs 7.50), and thiophenols (0.52 vs 3.18). BCL-XpKa and QupKake perform equivalently on primary and secondary amines, and QupKake outperforms BCL-XpKa on alinines (0.63 vs 0.44) and nitrogen aromatic heterocycles (0.63 vs 0.47) (Table 3). BCL-XpKaAcid had the highest error on aliphatic thiols (MAE = 1.72) and carbon acids (MAE = 3.38), which are poorly represented in TS-Acid (Figure 2A, B). Interestingly, the EO models outperformed BCL-XpKa on these sets of aliphatic thiols (1.51 vs 1.76), thiophenols (0.35 vs 0.40), and nitrogen aromatic heterocycles (0.63 vs 0.67). Predictions for each molecule can be found in Tables ST1–ST2.

**Table 3:** BCL-XpKa Mean Absolute Error (MAE) against Monoprotic Molecular Series with QM-Estimated p*K*_a_ Values by Thapa and Raghavachari^34^^a^

Series	BCL-XpKa LSO	BCL-XpKaAcid	EO-Acid	BCL-XpKaBase	EO-Base	QupKake
*Acids*						
Aliphatic Thiols	1.974	1.7587	1.5125	–	–	5.09
Benzoic Acids	0.4242	0.4105	0.6358	–	–	1.45
Carbon Acids	3.3846	3.2883	5.4239	–	–	–
Carboxylic Acids	1.0091	1.0193	1.0449	–	–	1.66
Phenols	0.6105	0.5912	1.1973	–	–	7.5
Thiophenols	0.5204	0.3996	0.3503	–	–	3.184
						
*Bases*						
Anilines	0.6294	–	–	0.6407	1.2209	0.438
Nitrogen Aromatic Heterocycles	0.6266	–	–	0.6654	0.6262	0.468
Primary Amines	0.4641	–	–	0.4178	0.6915	0.469
Secondary Amines 1	0.7995	–	–	0.5423	0.9668	0.578
Secondary Amines 2	0.4173	–	–	0.5241	0.6395	0.593

a BCL-XpKa LSO = Leave Set Out, where a restricted BCL-XpKa model was retrained on TS-Acid or -Base with the series of molecules withheld.

Beyond accurate p*K*_a_ prediction, it is often important in drug development to correctly predict the effects of small perturbations to a lead molecule’s ionizability.^35^ To assess BCL-XpKa’s sensitivity to such changes, we retrained BCL-XpKaAcid with the Oxygen-Acids set removed (many are present in Ch-Acid). Then, we identified 71 pairs of molecules in the Oxygen-Acids set that vary by a slight modification, such as the replacement of an amide with an ester. BCL-XpKa correctly predicts the direction of the p*K*_a_ change in 81.7% of these pairs (Figure 2C). To illustrate this effect, BCL-XpKa correctly predicts the complex impact of aromaticity on nitrogen basicity in a series of piperidine derivatives relevant to drug development. Introducing a neighboring phenyl group reduces predicted p*K*_a_ from 10.45 (true p*K*_a_ = 11.2) to 4.80 (5.00). Similarly, aromatization of piperidine to pyridine decreases predicted p*K*_a_ to 5.45 (5.20), and appending the same phenyl group to produce quinoline reduces p*K*_a_ to 4.54 (4.92) (Figure 2D). For acids, BCL-XpKa correctly predicts the inductive effect of electron-withdrawing groups on acidity using a series of phenol derivatives (Figure 2E). Here, fluorination at the ortho position increases acidity more than fluorination at the para position (8.74 vs 9.28), and substitution with multiple fluorine atoms has a greater effect than monosubstitution (7.36). While phenol was in our training data, the remaining molecules were not.

Together, these data, along with the variability of most models on the various commonly used benchmark test sets, suggest that these test sets are affected by sampling bias. Therefore, the field may benefit from larger common test sets of high-quality p*K*_a_ data.

### Atomic Sensitivity Analysis Provides Actionable, Atomic-Resolution Information on Model Predictions

**Figure 3:**
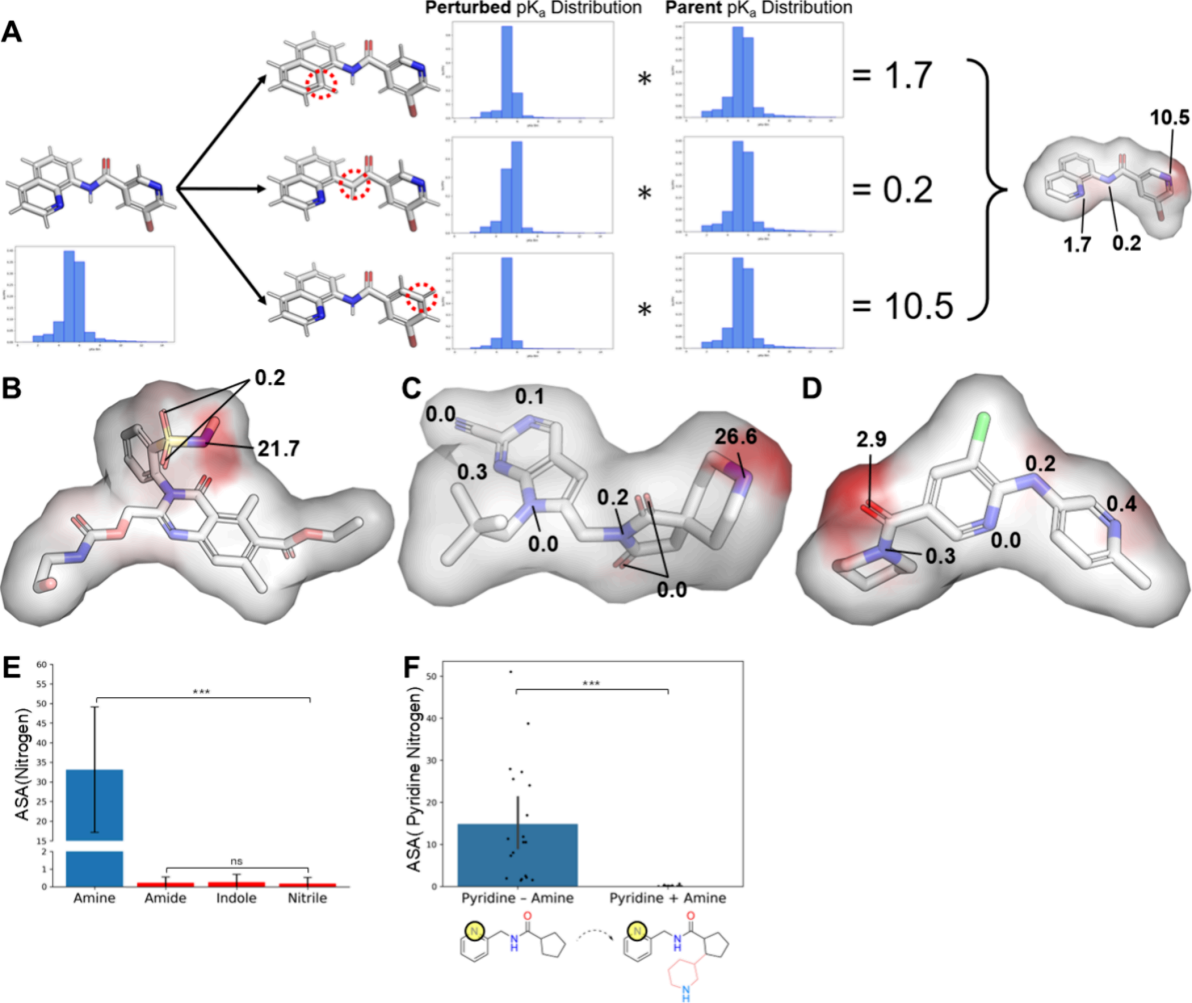
Atomic sensitivity for molecular analysis. (A) Schematic of the ASA protocol. Parent and Perturbed p*K*_a_ distributions refer to the discrete distributions output by BCL-XpKa. (B) Example ASA scores for an acid were scored by BCL-XpKaAcid. Here, the sulfonamide nitrogen atom is correctly selected as most acidic over other potentially acidic substructures. (C) Example ASA scores for a base were scored by BCL-XpKaBase. (D) A base in which the amide oxygen atom dominates ASA scores in the presence of more basic nitrogen atoms. This occurred in 4 of 61 amide-containing base compounds. (E) ASA scores of positive and negative control substructures for BCL-XpKaBase decomposition. Blue denotes the positive control, and red denotes the negative controls. (F) Modulation of pyridine nitrogen ASA score by addition of an amine group, with example shown below *x*-axis. ns = not significant. *** = *p* < 0.001.

Such atom-, substructure-, or pharmacophore-level interpretability of ML models in computational chemistry could accelerate computer-aided drug development on multiple fronts, from assisting in model training, to preparing and filtering molecules in virtual high-throughput screens, to guiding lead compound modification in lead optimization.^36^ To address this deficit, we developed atomic sensitivity analysis (ASA, Figure 3A), and we demonstrate its utility in decomposing BCL-XpKa’s predictions to assess model learning, identify ionization sites in complex small molecules, and guide lead optimization efforts by reducing molecular ionizability.

ASA compares an ML model’s prediction on a parent molecule before and after some perturbation. Here, we sequentially replace heteroatoms in the parent molecule with correctly hybridized carbons and then compare BCL-XpKa’s output p*K*_a_ probability distributions. The final ASA score is a scaled version of the KL-Divergence between these parent and perturbed p*K*_a_ distributions with coefficients used to minimize false positives and false negatives on a benchmark set (see Methods). As such, ASA relies on BCL-XpKa’s MTC architecture and Mol2D local-atomic environment descriptors. These descriptors are by definition more sensitive to single-atom changes in a molecule than typical fingerprints, and the discrete probability distribution provided for each molecule allows a more robust comparison of two molecules’ predictions than simply subtracting their predicted p*K*_a_ values output from an equivalent regression model. Note that ASA currently indicates only the magnitude of the change in p*K*_a_ and not the direction.

We benchmarked ASA on Novartis-Acid and Novartis-Base molecules with more than one potentially ionizable substructure (bases were allowed to have a single substructure only if they contained multiple potential atoms for protonation). The Novartis-Acid set (n = 98) had an average of 2.93 substructures/molecule, and ionization sites were most commonly in carboxylic acid (n = 37), sulfonamide (22), and phenol (13) derivatives. The Novartis-Base set (n = 102) had an average of 2.61 substructures/molecule, and ionization sites were most commonly in amine (n = 33), 2-aminopyrimidine (10), and 2-aminopyridine (8) derivatives.

For these sets, ASA ranged from 0–121.4. 1.5 was set as a cutoff for positive/negative prediction based on prior benchmarking during ASA’s creation. For acids, ASA had a sensitivity of 96.6%, specificity of 82.9%, and negative predictive value of 97.9% for selecting the most acidic atom. For bases, ASA had a sensitivity of 86.7%, specificity of 81.0%, and negative predictive value of 90.8% for selecting molecules’ most basic substructure. A molecule’s highest ASA score identified the most ionizable atom of 93.2% of acids and 73.5% of bases (Figure 3B, C).

Initially, we had hypothesized that ASA would highly score atoms that influenced p*K*_a_ but were not themselves ionizable. For example, performing ASA on an amide oxygen perturbs the amide (p*K*_a_ ∼ 16–18) into a vinyl amine (p*K*_a_ ∼ 8). Because of this significant change in p*K*_a_, we expected amide oxygen atoms to obscure the true ionization site. However, amide oxygen atoms were the dominant signal in only 4 of the 61 amide-containing bases evaluated (Figure 3D). While some nonionizable atoms did obscure results in this way, we hypothesize that a molecule’s remaining context supports ASA’s robustness to their influence (for example, see Figure 3C).

Finally, ASA scores were also consistent on a per-substructure basis. For example, free amines are the most basic group in 33.6% of the bases evaluated, and in each of these molecules, the amine nitrogen atom dominates the molecule’s ASA scores (33.1 ± 16.0). These scores were significantly higher than average scores for nitrogen atoms in amide (0.225 ± 0.332), indole (0.261 ± 0.444), and nitrile groups (0.180 ± 0.357) (*p* < 0.001), functional groups which are not traditionally ionizable at physiologic pH and which never contained the highest ASA atom (Figure 3E). That said, other substructures demonstrated consistent ASA scores when their basicity was not overshadowed by a much more basic group, and their ASA score was reliably ablated by the introduction of a significantly more basic amine group (Figure 3F, G).

### ASA Reveals BCL-XpKa Implicitly Learns Substructural Information Despite Local-Atomic Environment Descriptors

To investigate ASA scoring consistency further, we performed ASA on nitrogen atoms in the most frequently occurring substructures in the Novartis-Base set, beyond the controls discussed above. For each substructure, we compared molecules in which that substructure was the dominant driver of p*K*_a_ prediction (“dominant”) to molecules in which another substructure drove the p*K*_a_ prediction (“non-dominant”).

**Figure 4:**
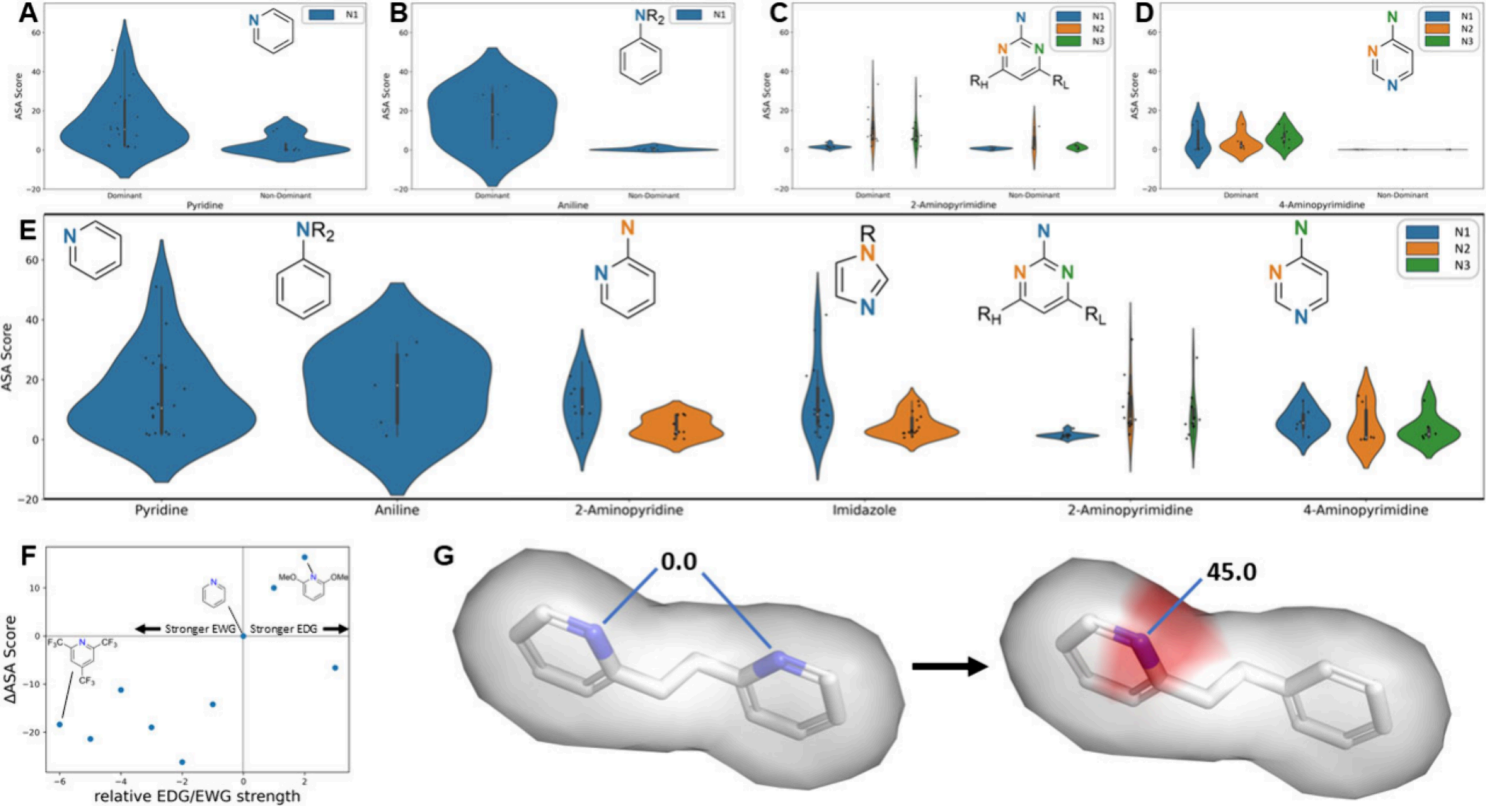
Atomic sensitivity analysis of substructures. (A–D) Violin plots of ASA scores for commonly occurring substructures when these substructures are the dominant site of a molecule’s ionization vs when a more dominant substructure was present. (E) Violin plots of ASA scores for commonly occurring substructures were obtained when these substructures were the dominant site of ionization. Notably, all test-set bases containing 2-aminopyridine and imidazole featured them as the dominant ionization site. (F) Influence of neighboring EWGs and EDGs on pyridine nitrogen’s ASA score, as measured by change in ASA (ΔASA = ASA_parent_ – ASA_mod_). Note that while ASA scores are strictly positive, an EWG reducing the ASA score of a pyridine nitrogen will make its ΔASA negative. (G) Masking effect of molecular symmetry on the ASA score. ASA = Atomic Sensitivity Analysis; EWG = electron-withdrawing group; EDG = electron-donating group.

All substructures examined demonstrated statistically significant loss of ASA signal when a more dominant substructure was present, indicating that the most dominant ASA atom significantly influences BCL-XpKa’s predicted p*K*_a_ distribution for the molecule (Figure 4A–D).

Strictly observing dominant substructures reveals that most substructures have consistent, substructure-specific trends in ASA scores (Figure 4E). This substructure-specificity even persists when separate substructures have nitrogen atoms with identical local atomic environments. For example, indole and imidazole both have a nitrogen bound to a hydrogen and two sp^2^ carbon atoms, but only imidazole is ionizable at physiologic pH values.^37,38^ While ASA correctly distinguishes that imidazole’s other nitrogen (which has a lone pair of electrons) is the most basic nitrogen in imidazole, it also scores the N–H nitrogen significantly higher than the identical motif in indole (Figure 3E), suggesting that this nitrogen is critical to imidazole’s observed basicity.

Some of these structures have surprisingly high variance in ASA scores, particularly in the pyridine and aniline substructures. From manual inspection, we hypothesized a portion of this variance is attributable to the impact of neighboring electron-donating and electron-withdrawing groups (EDGs, EWGs), which respectively increase and decrease basicity of the pyridine nitrogen atom. We tested this hypothesis by scoring manually created sets of pyridine derivatives with various EDG and EWG substituents and assessing ΔASA, the change in ASA score of the pyridine nitrogen atom from that of the unsubstituted parent structure. Indeed, this analysis demonstrates that neighboring EDGs tend to increase ASA scores, and neighboring EWGs tend to decrease ASA scores (Figure 4F). Importantly, symmetric substructures also contributed to this variance, as the effect of removing an atom during ASA scoring is masked by the persistent symmetric substructure (Figure 4G).

Together, these ASA findings suggest that BCL-XpKa has learned impressive substructural insights that are adaptable to the molecular context without directly encoding these substructures in the feature set.

### Atomic Sensitivity Analysis Can Inform Lead Compound Optimization in Drug Development

**Figure 5:**
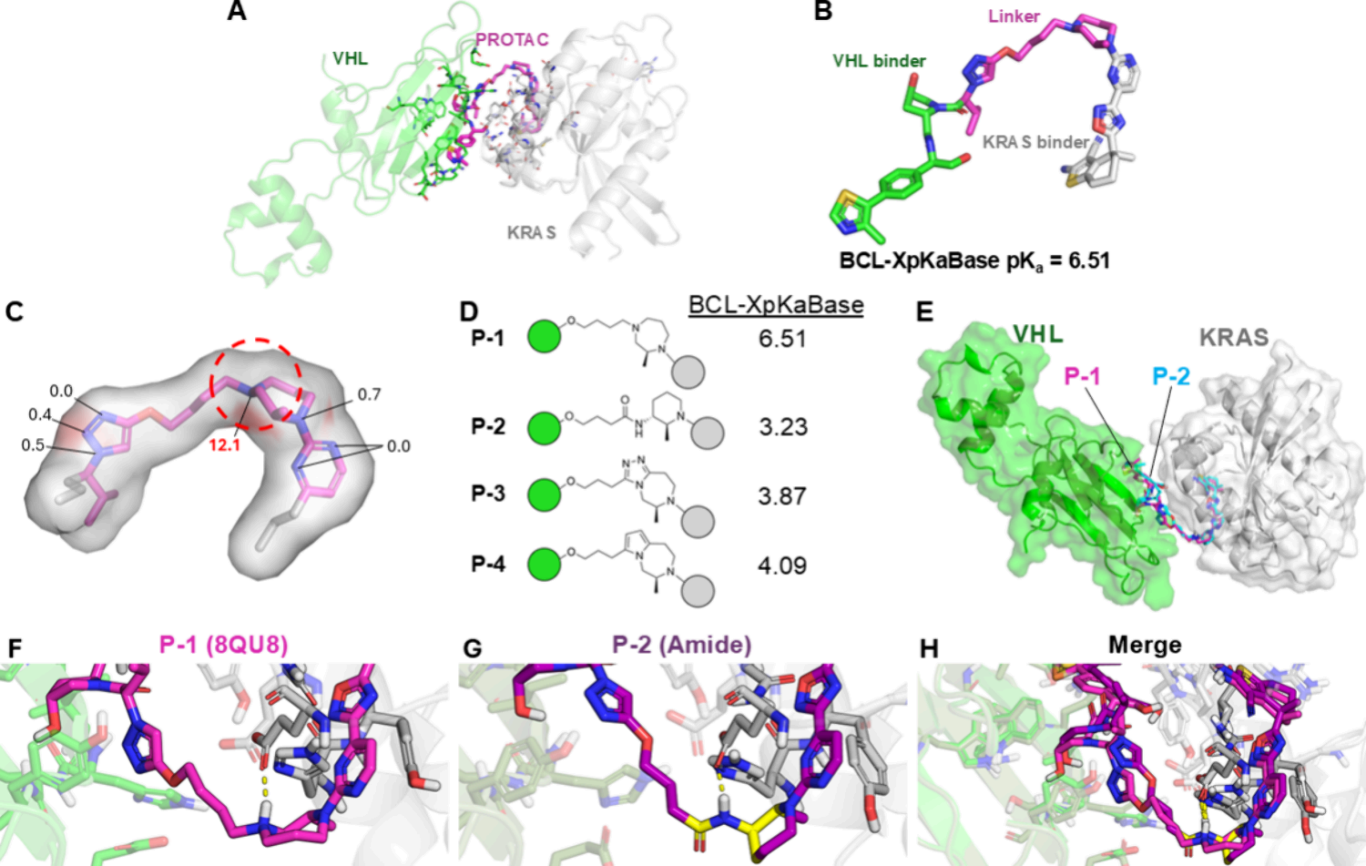
Atomic sensitivity for drug design. (A) Crystal structure (PDB: 8QU8) of pan-KRAS degrading PROTAC P-1 in the ternary complex with VHL and KRAS. (B) PROTAC P-1 is colored according to (A), with p*K*_a_ calculated by BCL-XpKaBase. (C) ASA scores for P-1 nitrogen atoms. VHL- and KRAS-binders were omitted for space. (D) Proposed bioisosteric P-1 linker modifications with p*K*_a_ values were predicted by BCL-XpKa. (E) A global view of ternary complex models of P-1 and P-2 docked into the VHL-KRAS PPI is identified in 8QU8. (F–H) Binding-site views of the 8QU8 crystal structure and the P-2 amide modification support similar PROTAC conformations that preserve the hydrogen bond to KRAS Q62. P-1 complex shown in brighter colors. P-2 complex shown in darker colors. P-2 linker modification highlighted in yellow.

Atomic sensitivity analysis also has a promising utility in prospective drug design. Significant interest has developed in the past two decades in targeted protein degradation via small-molecule Proteolysis Targeting Chimeras (PROTACs). PROTACs consist of two small molecules joined by a linker and form flexible ternary complexes with the target and an E3 Ligase, which allows for target ubiquitination and subsequent degradation by the 26S proteasome (Figure 5A, PDB: 8QU8). PROTACs degrade targets catalytically, making them an attractive strategy for challenging targets that have evaded small-molecule inhibition; however, PROTAC size and complexity plagues design efforts with poor bioavailability and cell permeability.^39^ These properties are generally optimized through modification of the PROTAC linker, as the ligand- and target-binding domains make specific contacts with their respective proteins. Here, we demonstrate how atomic sensitivities can guide rational changes to the PROTAC linker to minimize the PROTAC ionizability.

KRAS is a challenging target in oncology because it lacks a deep, well-defined pocket for small-molecule inhibition.^40^ Recently, Popow et al. created a KRAS-degrading PROTAC using the VHL E3 Ligase (Figure 5A, B).^41^ BCL-XpKaBase predicts that this PROTAC has a p*K*_a_ of 6.51 (P-1, Figure 4B). While this prediction is slightly lower than expected, it suggests that the PROTAC is protonatable at physiological pH. Atomic sensitivity analysis of the linker reveals one of two tertiary amines in P-1 drives this prediction (Figure 5C). In the crystal structure of the P-1 ternary complex, P-1 uses this protonated amine to form a salt bridge with KRAS Q62. While salt-bridge interactions promote strong drug binding, PROTACs only need to bind their targets transiently, and a protonatable amine is a liability for drug permeability. Based on this analysis, we evaluated several P-1 bioisosteric modifications at this amine that reduce predicted p*K*_a_ (Figure 5D). As expected, each PROTAC bioisosteric modification reduces the expected p*K*_a_. ASA demonstrates significant reduction of the targeted nitrogen (Figure S5). We next structurally characterized each modification’s ability to form ternary complexes with VHL and KRAS *in silico* using a well-established algorithm (Figure S6A, B). Here, we found that each modified PROTAC supports similar numbers of ternary complexes and that none prevents KRAS from being proximal to ubiquitin, a requisite for KRAS ubiquitination and degradation (Figure S6C).

Importantly, P-2 supports a similar VHL-KRAS TC pose to that identified in the 8QU8 crystal structure (ligand heavy atom RMSD < 2.5 Å, protein alpha-carbon RMSD < 10 Å). In this pose, amide-modified P-2 retains the ability to form a key hydrogen bond with KRAS Q62, for which P-1 utilizes its protonated tertiary amine (Figure 5F–H).

Together, these results demonstrate a computationally validated use of atomic sensitivities to guide lead-molecule optimization.

## Discussion

Here, we have presented BCL-XpKa, an open-source deep-learning-based p*K*_a_ predictor that embeds local atomic environments and reframes p*K*_a_ prediction as a classification problem while maintaining competitiveness with contemporary machine learning p*K*_a_ predictors, including GNNs and models trained via transfer learning on experimental data. We found that this multitask classification (MTC) approach directly informs the model’s uncertainty in each prediction, through which we identified molecular substructures in which BCL-XpKa had pathological confidence in its predictions (i.e., high confidence with high error). Beyond its absolute accuracy, BCL-XpKa reliably predicts the effects of common molecular modifications made to a hit/lead compound in a drug development program. We also showed that BCL-XpKa generalizes to foreign substructures better than equivalent models trained on MACCS- and Morgan Fingerprint-based descriptors via leave-substructural-class-out validation.

We then used BCL-XpKa as a model system to introduce atomic sensitivity analysis (ASA), a model interpretability tool which extracts the importance of each of a molecule’s atoms to BCL-XpKa’s predicted p*K*_a_ value for that molecule through direct perturbation of the input chemical structure. Other groups have successfully decomposed QSPR predictions in conceptually similar ways to ASA (e.g., removing atom information from a fingerprint, replacing an atom/substructure with a dummy one, using matched molecular pairs), but in general extracting atomic-level insights from models is difficult^18−21^ {Sushko, 2014 #52}. Even in GNNs, extracting node-level insight can be difficult due to the complexity of their internal molecular representations.^21^ However, ASA leverages BCL-XpKa’s encodings of local atomic environments, together with its characteristic output probability distributions, to produce reliable per-atom contributions, although room for improvement exists.

When applied to BCL-XpKa, ASA identifies the most ionizable atoms in both acids and bases with remarkable accuracy. ASA also revealed surprisingly consistent results for how BCL-XpKa considers ionizable substructures at the atomic level. These substructural ASA scores were responsive to neighboring electron-donating and -withdrawing groups, demonstrating that BCL-XpKa learns context-dependent substructural information from input local embeddings of atomic neighborhoods. Finally, we showed that pairing a QSPR model’s molecule-level predictions with atomic-level contributions is a powerful tool for guiding lead optimization using a published KRAS-degrading PROTAC. Here, BCL-XpKa and ASA directed linker modifications that reduced PROTAC ionizability while retaining critical PROTAC-KRAS contacts from the original crystal structure.

Several limitations exist in our current framework. First, while regression models can predict arbitrarily extreme values given enough quality data, BCL-XpKa must place all extreme values in two catch-all bins, “pK_a_ < 0” and “pK_a_ > 12”, given its MTC architecture. While this limits BCL-XpKa’s theoretical output range to −0.5 to 12.5, this is not consequential for biologically relevant pH scales and only marginally affects prediction accuracy. Second, BCL-XpKa is trained on a significant amount of predicted p*K*_a_ values from ChEMBL. While many existing p*K*_a_ predictors were trained with ChEMBL data due to the scarcity of ground-truth p*K*_a_ data, training ML models with synthetic data has significant known drawbacks (e.g., false confidence in incorrect predictions, error propagation of both systemic and individual errors, overfitting to model biases). To assess these risks in BCL-XpKa, we performed rigorous internal error testing with a leave-class-out approach, external testing against structurally diverse test sets, and compared BCL-XpKa against equivalent models trained exclusively with experimental data. We found that BCL-XpKa generalizes well to novel information, performs competitively on external sets, and outperforms the experimental-only models at nearly every task.

Further, ASA is currently limited to atomic-level model explainability. We demonstrate here that ASA has high sensitivity and specificity for selecting the most ionizable atom in a molecule. However, atoms can profoundly influence ionization without being directly ionized themselves (e.g., oxygen in a 1,2,4-oxadiazole vs imidazole). Though less frequently than expected, these types of atoms occasionally reduce ASA’s accuracy, representing an area of future improvement for ASA.

## Conclusions

BCL-XpKa and ASA have applications in computational chemistry generally as well as early- and late-stage drug development. As shown here, ASA scores can help scientists understand what a classification model has learned from a training set. This information can then guide training-set data augmentation or feature-set modifications. We believe this is especially important for p*K*_a_ prediction, where the amount of available data remains low.

Further, BCL-XpKa paired with ASA is positioned well to support high-quality small-molecule structure preparation for virtual high-throughput screening (vHTS). vHTS involves screening ultralarge libraries (ULLs) of small molecules (currently nearing 10^11^ molecules) for their ability to bind to a protein target. vHTS has notoriously low hit rates, and improper protonation of ULL molecules can contribute to both false-positive and false-negative vHTS screens. BCL-XpKa and ASA’s speed and accuracy at predicting p*K*_a_ and identifying ionization sites in multiprotic species make this tool a valuable asset for ULL structure preparation and downstream protein–ligand analysis in vHTS. We are actively working to incorporate ASA as a protonation tool within the BCL.

Finally, as demonstrated here, BCL-XpKa paired with ASA can identify ionizable regions in a compound for modification in hit-to-lead or lead optimization. In the future, applying ASA to predictors of ADMET/DMPK may facilitate understanding of important but difficult-to-interpret liabilities in a hit or lead compound.

## Methods

### Data Set Availability

All molecules used in this work, together with a description of where each set is used, can be found in the Extended Data.

### Training Data sets

ChEMBL27 is an open-source database contains over 2 million molecules with various physicochemical descriptors.^42^ ACDlabs was used to calculate acidic p*K*_a_ and basic p*K*_a_ values (chembl_acid_pka and chembl_base_pka, respectively) for ChEMBL molecules, and molecules that were included in our test sets were excluded.^29^ We also generated negative data (molecules with no ionization site) in the BCL and set chembl_acid_pka = 50 and chembl_base_pka = −50. In sum, acidic p*K*_a_ models were trained on 988,643 molecules, and basic p*K*_a_ models on 812,918 molecules. When test sets included training-set molecules, models were retrained with the test-set molecules withheld prior to evaluation.

### Synthetic Molecule Generation for Data Set Augmentation

The acid training set was significantly imbalanced at physiologically relevant p*K*_a_ values, an issue that has been noted by other groups using the ChEMBL data set.^9,11^ To mitigate this issue, we augmented the data set at these p*K*_a_ values as follows. First, we randomly generated hundreds of thousands of druglike molecules with ionizable functional groups within the BCL. Then, we filtered this synthetic set by consensus scoring between the imbalanced BCL-XpKaAcid prediction and ACD/Laboratories Percepta prediction (i.e., molecules with | pKaBCL – pKaACD | > 0.5 were discarded). The final impact of this acid-set augmentation on the TS-Acid p*K*_a_ distribution can be seen in Figure S1A. Augmenting the acid training set with synthetic, ionizable data did not affect aggregate model performance on the Novartis-Acid set (0.81 balanced versus 0.82 imbalanced). The base training set was already well-balanced without augmentation of the data beyond the negative data.

During model evaluation, we identified that deprecated BCL-XpKa predicted highly inaccurate p*K*_a_ values for small molecules with no ionization sites. For example, it predicted benzene to have a p*K*_a_ value of 6.56, potentially due to the relevance of aromaticity or *sp*^2^ carbons to ionization. To account for this error, we created a set of 120,000 nonionizable hydrocarbon and heteroatom-containing small molecules. We assigned these molecules a p*K*_a_ value of 50 for BCL-XpKaAcid and a low p*K*_a_ value of −5 for BCL-XpKaBase, reflecting that these molecules would only be ionized at nonbiological pH values. We benchmarked several augmented training sets with increasing amounts of negative data, finding that 60K nonionizable molecules corrected this pathological behavior and slightly improved performance on the Novartis sets for both acid (MAE = 0.79 augmented vs 0.82 original) and base (0.86 vs 0.93) models, whereas inclusion of all 120 K molecules decreased model performance.

### Molecule Preparation

Molecular 3D structures were standardized using Corina for training and testing BCL-XpKa.^43^ For external models, structure preparation followed the authors’ direction, and Corina was used if no structure preparation method was mentioned. This standardization was used exclusively for downstream usability, as BCL-XpKa solely uses 2D descriptors. All PROTAC modifications introduced in Figure 5 were minimized in the Molecular Operating Environment (MOE) prior to ternary complex ensemble generation.

### Molecular Features

The Mol2D molecular descriptor set was used to encode molecules, as described elsewhere.^22^ Briefly, for each atom in a molecule, Mol2D encodes information about that atom, the bonds made to that atom, and the atoms one bond length away from that atom.

To train a multitask classifier with N output labels, ChEMBL p*K*_a_ values were encoded in an *N* x 1 result label , where the first entry corresponds to p*K*_a_ < 0, the last entry to p*K*_a_ ≥ 12, and the *i*th entry to (12/*N*) × (*i* – 1) ≤ p*K*_a_ < (12/*N*) × *i*. For regression models, p*K*_a_ in the ChEMBL set was used directly as the result label.

### Model Training and Validation

Artificial neural networks were built in C++ for the Biology and Chemistry Library (BCL), an open-source cheminformatics platform created and maintained by our lab. Each model was trained for 250 iterations without early stopping, which our lab previously found to be unnecessary when dropout is used.^44^ An upper bound for model performance was calculated through random-split cross validation. A lower-bound for performance was calculated through leave-class-out cross validation (LCO–CV), in which the training set was divided into 30 subset {*C*_*i*_}_*i* = 1_^30^ based on ionizable groups defined in literature.^45^ Thirty models were then trained in an iterative all-but-one scheme. Model internal performance was evaluated using the logarithmic receiver operating characteristic curve (logAUC) and AUC using the BCL model:ComputeStatistics application.

### Model Output and Evaluation

MTCs with N output labels calculate N local positive predictive values (localPPV), where the *i*th localPPV denotes the probability that p*K*_a_ lies in the *i*th p*K*_a_ interval (see Molecular Features above). For model evaluation, we report mean absolute error (MAE) for reader familiarity. In the Supporting Information, we also provide a Brier score for each model, which is a proper scoring rule^a^ for more rigorous evaluation of classification model output.

For each molecule *m*_*i*_ ∈ *Y* in a test set *Y* with *M* molecules, the p*K*_a_ of *m*_*i*_ was encoded with a binary result label  as described above. MTC scored each molecule, providing a discrete probability distribution *P*_*i*_ describing the molecule’s likely p*K*_a_ interval membership. From these distributions, MAE was calculated as
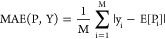
where *E*[*P*_*i*_] is the expected value of *P*_*i*_ (i.e., a weighted sum of the median p*K*_a_ value of each bin multiplied by the probability that the molecule’s p*K*_a_ value lies in that bin). Similarly, for several models a Brier score was calculated as
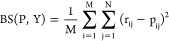
where *r*_*ij*_ ∈ *R*_*i*_ is the *j*th result label for the *i*th molecule in the test set (i.e., whether its p*K*_a_ value lies in the *j*th p*K*_a_ interval), and *p*_*ij*_ is the localPPV that the *i*th molecule’s p*K*_a_ value lies in the *j*th p*K*_a_ interval.

Throughout, MAE is used to compare MTC to regression and MTC to MTC models. Percent accuracy of categorization is not included as it is an improper and discontinuous scoring metric.

### Atomic Sensitivity Analysis

Atom replacement schemes were coded in C++ within the BCL. A parent molecule *m* is scored by BCL-XpKa to produce P, a discrete probability distribution of potential p*K*_a_ values. Heteroatom *a* in the parent molecule is replaced with an appropriately hybridized carbon atom, and the perturbed molecule is rescored to produce P_*a*_^′^. The dissimilarity between these distributions was calculated by their Kullback–Leibler (KL) divergence:
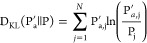
where P_j_ and P_*a*,j_^′^ are localPPVs as described in Model Output and Evaluation. Briefly, the KL divergence of these two probability distributions is best interpreted as the relative entropy between these distributions, where D_KL_(P′||P) = 0 denotes that the distributions are identical (there would be no “surprises” if a given sample came from P vs P′), and higher values denote more dissimilarity. Finally, KL divergences were empirically denoised using molecules that contained one ionizable substructure^45^ per molecule to generate ASA scores:
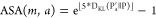


This formula was gradually adapted to reduce false-positive reporting, as follows. The floor function zeroes out the ASA score of perturbations that only marginally modify the output p*K*_a_ distribution. Exponentiation increases the separation between true positive and false positive data (e.g., the Mean Value Theorem on *f*(*x*) = *e*^*x*^ demonstrates that *e*^*a*^ – *e*^*b*^ > *a* – *b* for any real numbers *a*,*b* > 1). We subtract 1 because *e*^0^ = 1. Lastly, the “5” coefficient was chosen based on a small benchmark set for similar signal-noise processing and is by no means a proven optimal coefficient.

### PROTAC Ternary Complex Ensemble Generation

Ternary complexes (TCs) were constructed according to Drummond et al.^46^ Briefly, protein–protein interactions (PPIs) with the PROTAC binding pockets near each other, as well as a set of up to 10000 PROTAC conformations, were produced in the Molecular Operating Environment (MOE). PROTAC conformations were then docked into the PPIs and filtered according to the authors’ criteria. TCs were then clustered on both protein- and PROTAC-conformations to produce “double clusters.” Protein-conformational clustering was done at CA-RMSD < 10 Å. PROTAC clustering was done at heavy-atom RMSD < 2.5 Å.

### Hardware

All models were trained with 18 Intel Xenon W-2295 CPU cores. PROTAC TC formation was performed in part by using an Nvidia RTX A5000 GPU.

## Data Availability

Statement BCL-XpKa and ASA are found in the molecule:Properties and cheminfo:MoleculeFeatures apps, respectively, within the BCL codebase located at https://github.com/decortja/BCL_for_XpKa-Manuscript. All data sets used for model training and evaluation, the descriptor set used for molecule embedding, and example implementations of BCL-XpKa and ASA are available at 10.5281/zenodo.13336698 and described in the Supporting Information.

## Supporting Information Available

Sets of molecules used for training and testing the final BCL-XpKaAcid and BCL-XpKaBase models. Descriptor set: BCL-XpKaAcid (Training set: final_training_set_acids.sdf.gz; Test set 1: test-set_NovartisAcids.sdf; Test set 2: test-set_HaiyingOxygenAcids.sdf). BCL-XpKaBase (Training set: final_training_set_bases.sdf.gz; Test set: test-set_NovartisBases.sdf). Evaluation of model hyperparameters: Table S1. PDB 8QU8 used for computational PROTAC optimization in Figure 5 (PDF)
UMol2D descriptor set used to embed molecules for all models presented in this paper (XLSX)

## Footnotes

a “Proper scoring rule” is a term in statistics for a loss function that is minimized if the probability distribution output from the model is identical to the ground-truth probability distribution. When the bidirectional holds, the scoring rule is further labeled a *strictly proper* scoring rule.
