# Supplementary material for: Interpretable Deep-Learning pKa Prediction for Small Molecule Drugs via Atomic Sensitivity Analysis
Source: J Chem Inf Model. 2024 Dec 30;65(1):101–13. doi: 10.1021/acs.jcim.4c01472 (PMC11733947; doi:10.1021/acs.jcim.4c01472)
Supplement: Supplementary file 1 — ci4c01472_si_002.pdf [file ci4c01472_si_002.pdf]

Supplementary Information to "Interpretable deep-learning pKa prediction for small molecule drugs via atomic sensitivity analysis"

Authors: Joseph DeCorte[1-3*], Benjamin Brown[4,5], Jeffrey Rathmell, Jens Meiler[1,2,4-5,7]

*Corresponding author. Email: joseph.a.decorte@vanderbilt.edu

TABLE OF CONTENTS

Supplementary Figure S1: pKa ranges for BCL-XpKaAcid and BCL-XpKaBase training sets………………2

Supplementary Figure S2: Physicochemical properties of TS-Acid……………………………………………..3

Supplementary Figure S3: Physicochemical properties of TS-Base……………………………………………4

Supplementary Figure S4 BCL-XpKa prediction confidence versus accuracy……………………………...5

Supplementary Figure S5: ASA analysis for BCL-XpKaBase applied to all PROTAC modifications made in Figure 5………………………………………………………………………………..6

Supplementary Figure S6: Structural analysis of the PROTAC modifications described in Figure 5…………………………………………………………………………………………..………7

Supplementary Table ST1: BCL-XpKaAcid predictions on acidic molecular series from Thapa and Rhagavachari……………………………………………………………………8

Supplementary Table ST2: BCL-XpKaBase predictions on basic molecular series from Thapa and Rhagavachari…………………………………………………………………..11

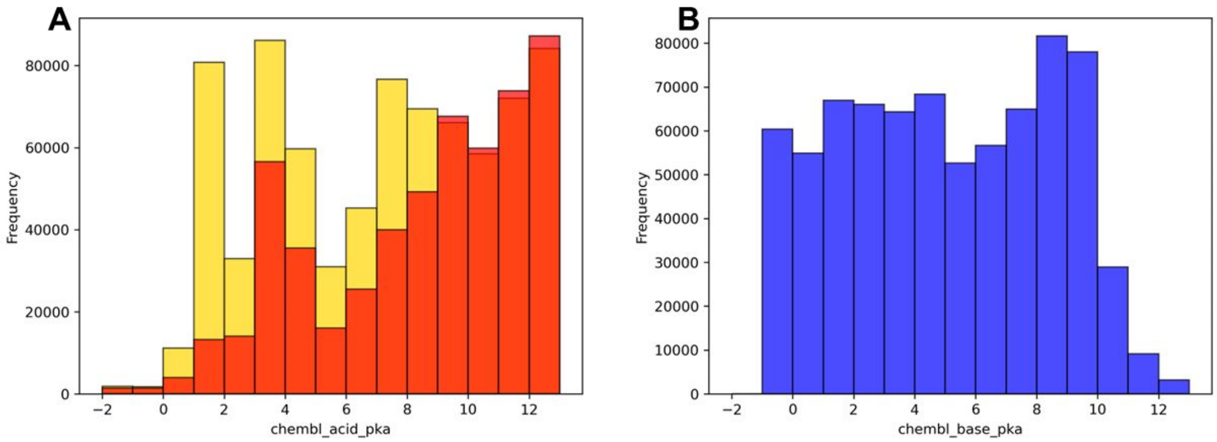

Supplementary Figure S1: pKa ranges for BCL-XpKaAcid and BCL-XpKaBase training sets. A-B: pKa range of TS-Acid (**A**) and TS-Base (**B**), the datasets used to train BCL-XpKaAcid and Base, respectively. Gold bars in (**A**) denote dataset augmentation performed within the BCL to balance the set a bit better (see *Methods*). Note that each has 60,000 nonionizable molecules that are not displayed here. These negative molecules were assigned a chembl_acid_pka of 50, and a chembl_base_pka of -50, which are binned into BCL-XpKa's extreme bins.

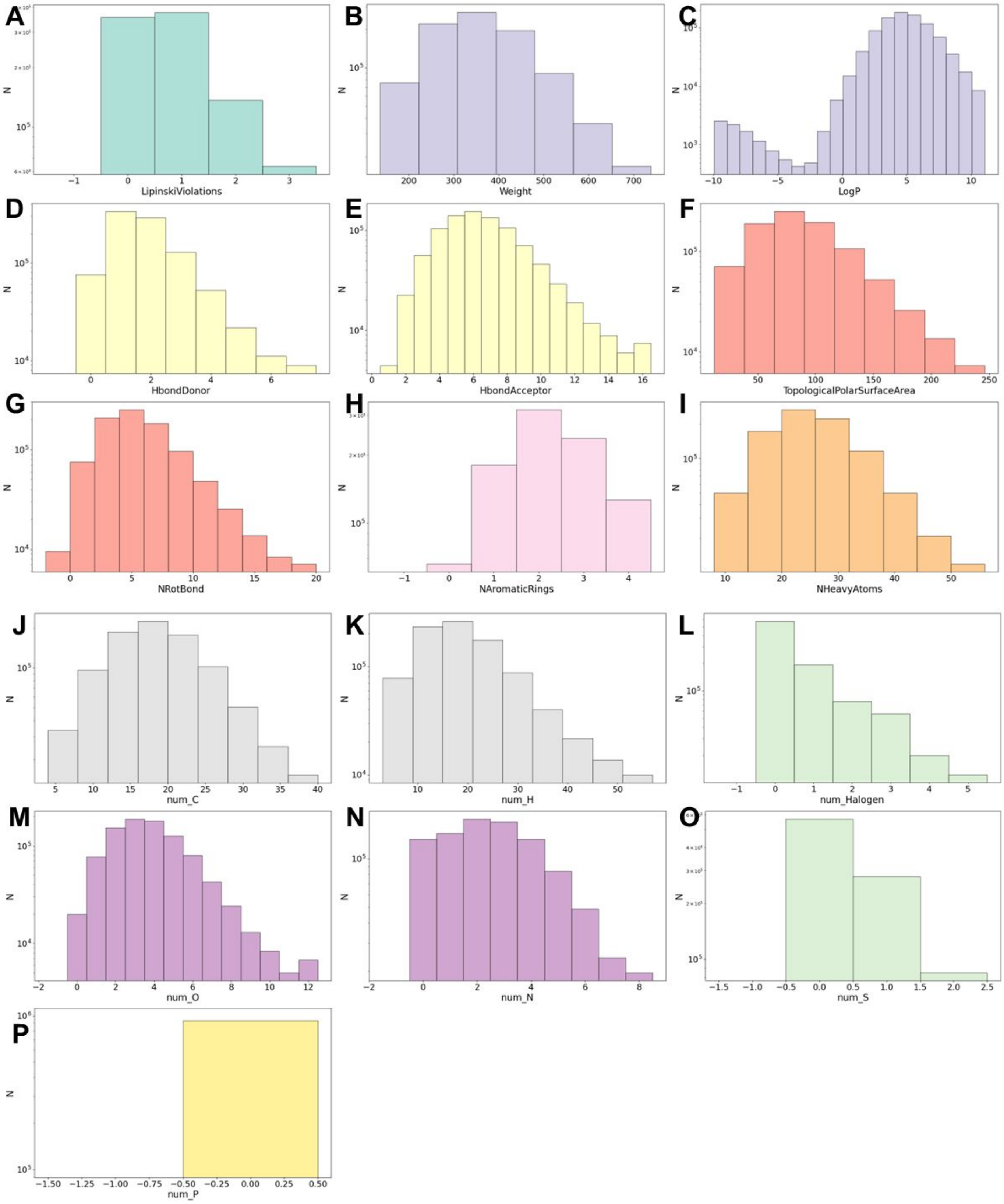

Supplementary Figure S2: Physicochemical properties of TS-Acid. **A**: LipinskiViolations is the total number of Lipinski Rule of Five violations in a molecule. **B:** Molecular Weight. **D-E:** the number of Hydrogen Bond Donors and Acceptors per molecule. **G:** NRotBond is the number of rotatable bonds in a molecule. **J-P**: Counts of specific atoms in each molecule.

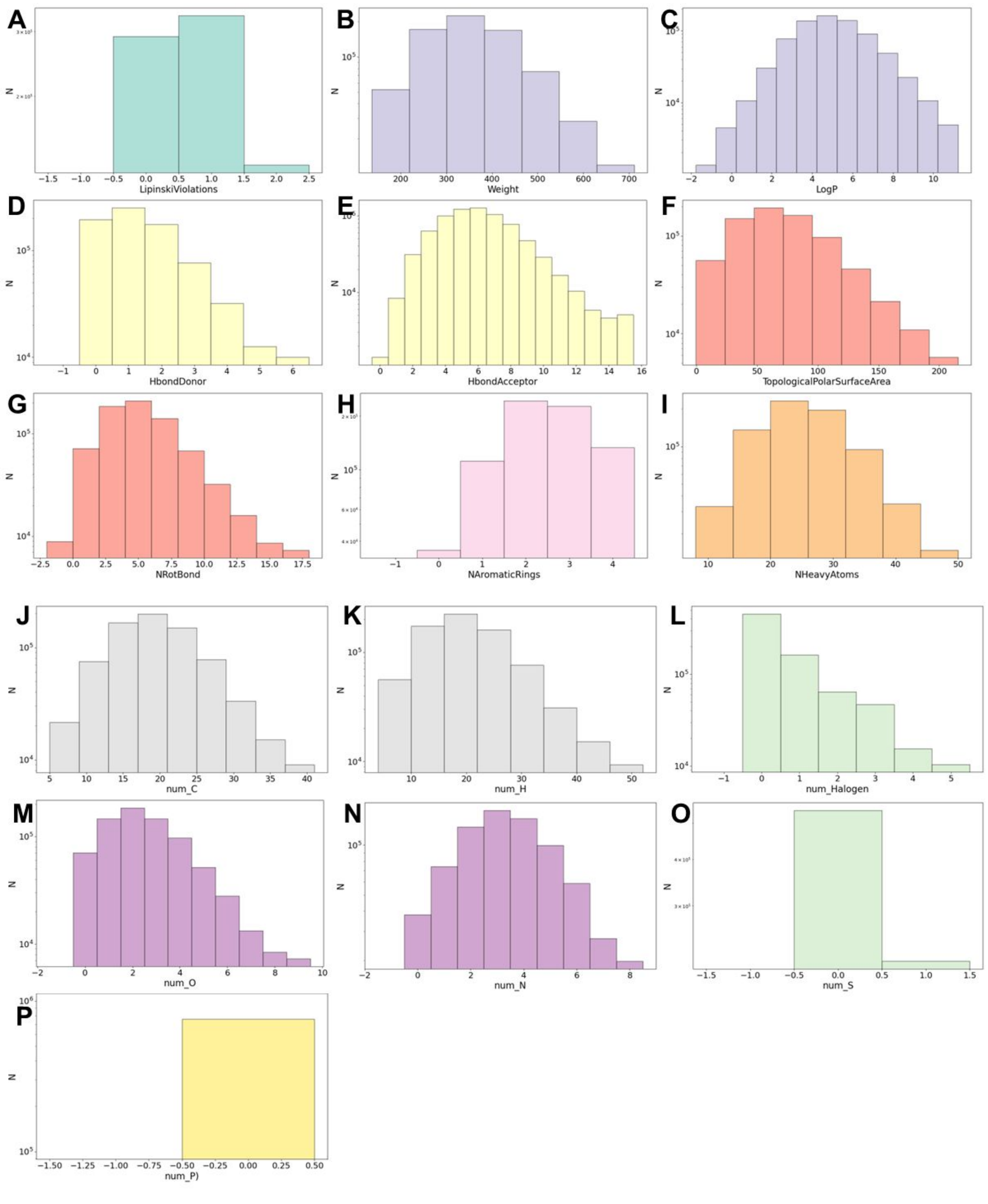

Supplementary Figure S3: **Physicochemical properties of TS-Base**. **A**: LipinskiViolations is the total number of Lipinski Rule of Five violations in a molecule. **B:** Molecular Weight. **D-E:** the number of Hydrogen Bond Donors and Acceptors per molecule. **G:** NRotBond is the number of rotatable bonds in a molecule. **J-P**: Counts of specific atoms in each molecule.

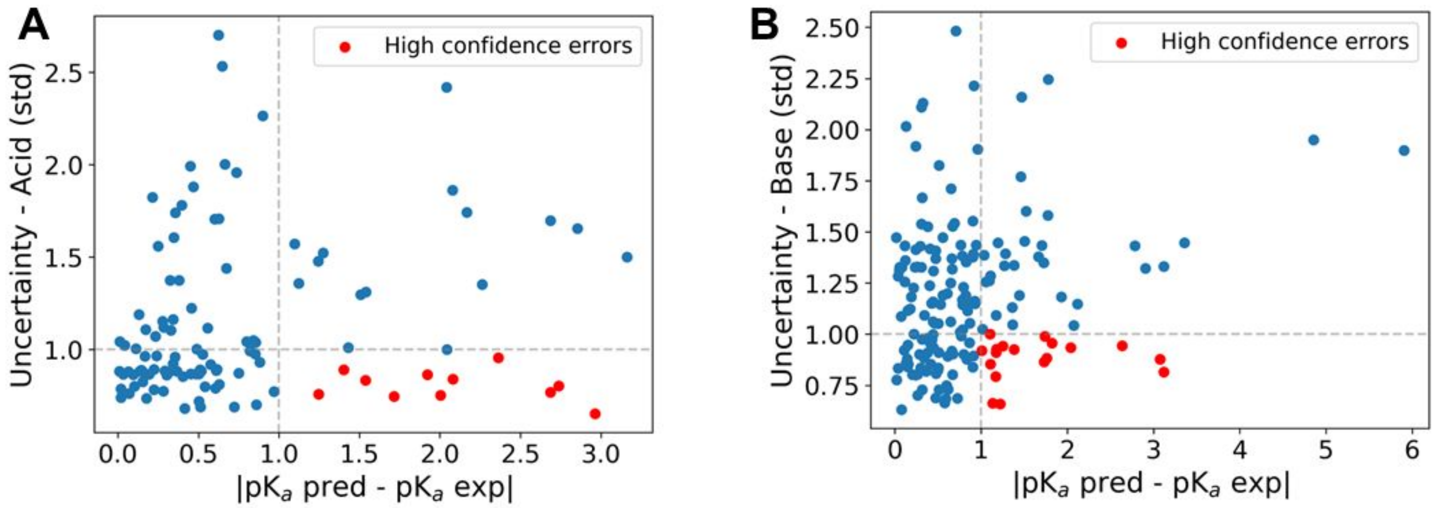

Supplementary Figure S4 BCL-XpKa prediction confidence versus accuracy. **A-B**: Performance of BCL-XpKaAcid (**A**) and BCL-XpKaBase (**B**) on the Novartis-Acid and Novartis-Base sets, respectively. The error of each prediction is provided against the standard deviation of BCL-XpKa's predicted pK$_a$ distribution for each molecule. Red data indicate predictions where BCL-XpKa was confident in its prediction (σ < 1.0), yet the error exceeded 1 pK$_a$ unit.

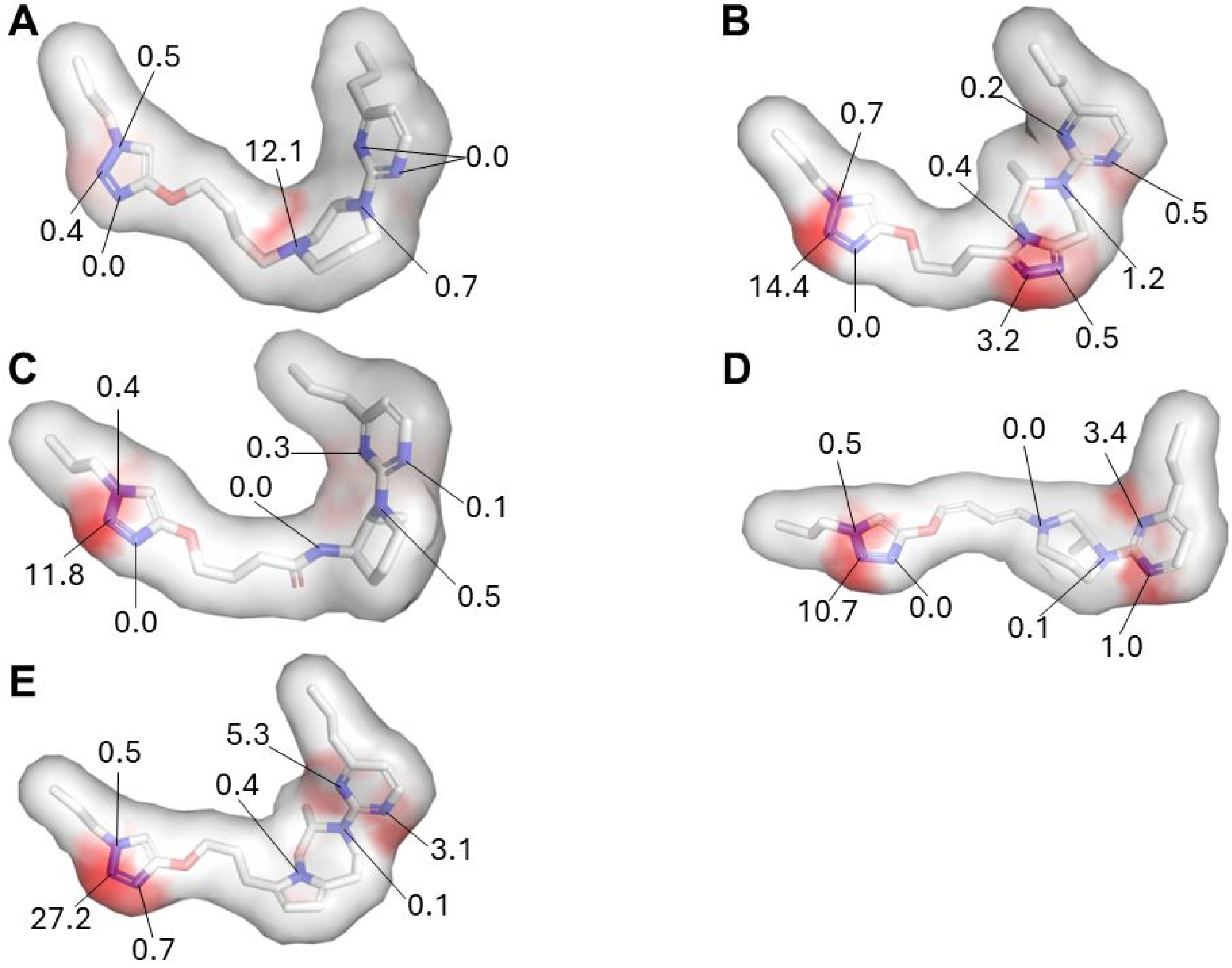

Supplementary Figure S5: ASA analysis for BCL-XpKaBase applied to all PROTAC modifications made in Figure 5. A,B,C,E: The PROTACs described in the main text. **D**: A structural negative-control PROTAC that is too rigid to produce many productive ternary complexes with VHL and KRAS.

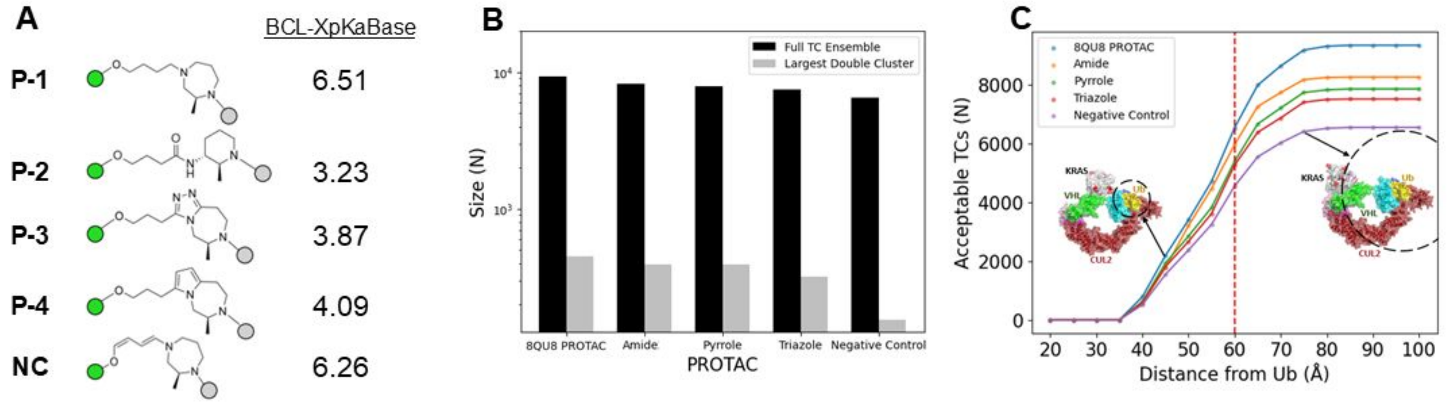

Supplementary Figure S6: Structural analysis of the PROTAC modifications described in Figure 5. **A**: each PROTAC with its structure and pK_a predicted by BCL-XpKaBase. **B**: The number of ternary complexes (black) and the size of the largest structural double-cluster, clustered by protein alpha-Carbon RMSD and PROTAC heavy-atom RMSD. **C**: A cumulative distribution of the ternary complex as a function of distance from Ubiquitin. 60 Angstroms set as a literature-identified cutoff for productive ternary-complex formation.

Model	Series	pKa_predicted	pKa_experimental	SMILES
Acid	aliphatic_thiols	12.41026347	7.86	C=CCS
Acid	aliphatic_thiols	11.89998737	7.95	SCC(=O)OCC
Acid	aliphatic_thiols	9.961065301	8.62	SC[C@@H](CO)S
Acid	aliphatic_thiols	11.67710343	9.38	C(OCC)CS
Acid	aliphatic_thiols	9.606174663	9.72	OCCS
Acid	aliphatic_thiols	9.308216553	9.85	SC(C)(C)CO
Acid	aliphatic_thiols	12.41026347	9.96	C(=C)CS
Acid	aliphatic_thiols	4.476618968	10.27	C(C(=O)[O])CS
Acid	aliphatic_thiols	12.1952806	10.33	SC
Acid	aliphatic_thiols	11.92187125	10.61	CCS
Acid	aliphatic_thiols	11.9617243	10.67	C(CC)CS
Acid	aliphatic_thiols	11.7773299	10.86	SC(C)C
Acid	aliphatic_thiols	10.47397804	11.05	SC(C)(C)C
Acid	aliphatic_thiols	10.58347071	11.22	SC(C)(C)CC
Acid	benzoic_acids	3.479663979	2.17	c1ccc(c(c1)N(=O)=O)C(=O)O
Acid	benzoic_acids	3.842201498	2.94	Clc1ccccc1C(=O)O
Acid	benzoic_acids	3.273604686	2.95	c1ccc(c(c1)C(=O)O)C(=O)O
Acid	benzoic_acids	2.649897008	2.98	c1ccc(c(c1)O)C(=O)O
Acid	benzoic_acids	3.783684995	3.27	c1ccc(c(c1)F)C(=O)O
Acid	benzoic_acids	3.899352801	3.45	c1ccc(cc1N(=O)=O)C(=O)O
Acid	benzoic_acids	4.390322142	3.46	c1(c(cccc1)C(=O)O)C(C)(C)C
Acid	benzoic_acids	4.065588168	3.51	c1(ccc(cc1)C(=O)O)C(=O)O
Acid	benzoic_acids	3.494587743	3.54	c1c(cc(cc1)C(=O)O)C(=O)O
Acid	benzoic_acids	4.508196803	3.77	c1(ccccc1C(=O)O)CC
Acid	benzoic_acids	4.308918881	3.83	c1ccc(cc1Cl)C(=O)O
Acid	benzoic_acids	4.033263294	3.87	c1ccc(cc1F)C(=O)O
Acid	benzoic_acids	4.508465147	3.91	c1ccc(c(c1)C)C(=O)O
Acid	benzoic_acids	4.494999917	3.99	c1(ccc(cc1)C(=O)O)Cl
Acid	benzoic_acids	3.753468495	4.08	c1ccc(cc1O)C(=O)O
Acid	benzoic_acids	3.550049441	4.09	c1ccc(c(c1)OC)C(=O)O
Acid	benzoic_acids	3.89428881	4.09	c1cc(cc(c1)OC)C(=O)O
Acid	benzoic_acids	4.510608242	4.14	c1(ccc(cc1)C(=O)O)F
Acid	benzoic_acids	3.829941376	4.17	c1(cccc(c1)C(=O)O)OCC
Acid	benzoic_acids	3.562200186	4.21	c1(ccccc1C(=O)O)OCC
Acid	benzoic_acids	4.50823151	4.24	c1c(cc(cc1)C(=O)O)C
Acid	benzoic_acids	4.53205639	4.34	c1(ccc(cc1)C(=O)O)C
Acid	benzoic_acids	4.545438543	4.35	c1(ccc(cc1)C(=O)O)C(C)C
Acid	benzoic_acids	4.545678735	4.35	c1(ccc(cc1)C(=O)O)CC
Acid	benzoic_acids	4.533288202	4.45	c1(ccc(cc1)C(=O)O)OCC
Acid	benzoic_acids	4.52775494	4.47	c1c(ccc(c1)OC)C(=O)O
Acid	benzoic_acids	4.454937817	4.58	c1cc(ccc1O)C(=O)O
Acid	benzoic_acids	4.423833299	4.92	c1(ccc(cc1)C(=O)O)N(=O)=O
Acid	carbon_acids	12.19924322	9.9	c1ccc2c(c1)c1c([C@H]2C(=O)C)cccc1
Acid	carbon_acids	12.3073739	10.4	C(=O)[C@H](c1ccccc1)c1ccccc1
Acid	carbon_acids	12.39932002	10.5	c1ccc2c(c1)c1c([C@H]2C(=O)SC)cccc1

Acid	carbon_acids	12.27343511	11.5	c1ccc2c(c1)c1c([C@H]2C(=O)OC)cccc1
Acid	carbon_acids	12.27986072	13.1	C(=O)Cc1ccccc1
Acid	carbon_acids	12.41346116	14.8	C(=O)(CNC(=O)C)c1ccc(cc1)C
Acid	carbon_acids	12.39214263	16.7	C(=O)C
Acid	carbon_acids	12.44307987	18.3	C(=O)(C)c1ccccc1
Acid	carbon_acids	12.44348203	19.2	C(=O)(C)c1ccc(cc1)C
Acid	carbon_acids	12.46133834	19.3	C(=O)(C)C
Acid	CarboxylicAcids	4.06370467	-0.26	C(=O)(O)C(F)(F)F
Acid	CarboxylicAcids	3.439783388	0.65	C(=O)(O)C(Cl)(Cl)Cl
Acid	CarboxylicAcids	3.422992226	1.24	C(=O)(O)C(F)F
Acid	CarboxylicAcids	3.801663532	1.3	C(=O)(O)C(Cl)Cl
Acid	CarboxylicAcids	3.459184015	2.44	C(#N)CC(=O)O
Acid	CarboxylicAcids	4.432351105	2.66	C(=O)(O)CF
Acid	CarboxylicAcids	4.493264415	2.8	C(=O)(O)[C@@H](Cl)C
Acid	CarboxylicAcids	4.376495556	2.81	C(=O)(O)CCl
Acid	CarboxylicAcids	4.0359432	2.86	C#CCC(=O)O
Acid	CarboxylicAcids	3.776995656	2.86	C(=O)(O)CBr
Acid	CarboxylicAcids	4.393080392	3.07	C(=O)(O)CC(F)(F)F
Acid	CarboxylicAcids	3.964241668	3.53	C(=O)(O)CC(=O)C
Acid	CarboxylicAcids	3.764392866	3.54	COCC(=O)O
Acid	CarboxylicAcids	4.376737299	3.75	C(=O)O
Acid	CarboxylicAcids	3.348545848	3.83	C(=O)(O)CO
Acid	CarboxylicAcids	3.535311407	3.87	C(=O)(O)[C@H](O)C
Acid	CarboxylicAcids	4.497315293	4.1	C(CC(=O)O)Cl
Acid	CarboxylicAcids	4.474698593	4.26	C(=O)(O)C=C
Acid	CarboxylicAcids	4.503367662	4.31	c1ccccc1CC(=O)O
Acid	CarboxylicAcids	4.502977721	4.35	C=CCC(=O)O
Acid	CarboxylicAcids	4.494511092	4.52	ClCCCC(=O)O
Acid	CarboxylicAcids	4.499409009	4.76	C(=O)(O)C
Acid	CarboxylicAcids	4.513557815	4.82	C(=O)(O)CCC
Acid	CarboxylicAcids	4.49716911	4.87	C(=O)(O)CC
Acid	CarboxylicAcids	4.510024543	4.9	C1CCC(CC1)C(=O)O
Acid	CarboxylicAcids	4.4942458	5.05	C(=O)(O)C(C)(C)C
Acid	phenols	8.244550568	6.79	c1cc(c(cc1)O)C=O
Acid	phenols	7.986039851	7.14	c1(ccc(cc1)O)N(=O)=O
Acid	phenols	7.272469095	7.23	c1ccc(c(c1)N(=O)=O)O
Acid	phenols	8.724416138	7.66	c1c(ccc(c1)O)C=O
Acid	phenols	8.505212902	7.95	c1c(ccc(c1)O)C#N
Acid	phenols	9.187526979	8	c1c(cc(cc1)O)C=O
Acid	phenols	7.660892345	8.35	c1ccc(cc1N(=O)=O)O
Acid	phenols	8.788370302	8.41	c1cc(ccc1O)C(=O)OC/C=C/C
Acid	phenols	8.688424045	8.47	c1cc(ccc1O)C(=O)OC
Acid	phenols	8.587928468	8.47	c1cc(ccc1O)C(=O)OCCCC
Acid	phenols	8.680243967	8.48	Clc1ccccc1O
Acid	phenols	8.659500525	8.5	c1cc(ccc1O)C(=O)OCC
Acid	phenols	8.362735642	8.61	c1cc(cc(c1)O)C#N

Acid	phenols	8.764534955	8.81	c1ccc(c(c1)F)O
Acid	phenols	9.302790563	9.02	c1ccc(cc1Cl)O
Acid	phenols	9.48477598	9.28	c1ccc(cc1F)O
Acid	phenols	9.298632938	9.38	c1(ccc(cc1)O)Cl
Acid	phenols	4.436774051	9.39	c1(ccc(cc1)O)C(=O)[O]
Acid	phenols	9.654586875	9.44	c1ccc(cc1O)O
Acid	phenols	9.5543255	9.48	c1ccc(c(c1)O)O
Acid	phenols	9.71490969	9.51	c1c(ccc(c1)c1ccccc1)O
Acid	phenols	9.672889539	9.59	c1cc(cc(c1)c1ccccc1)O
Acid	phenols	9.755693579	9.65	c1ccc(cc1OC)O
Acid	phenols	10.05342227	9.82	c1cc(ccc1O)CO
Acid	phenols	9.794197613	9.83	c1cc(cc(c1)O)CO
Acid	phenols	10.44111799	9.9	c1cc(cc(c1)O)CC
Acid	phenols	9.72822534	9.92	c1cc(c(cc1)O)CO
Acid	phenols	9.694515623	9.93	c1ccc(c(c1)c1ccccc1)O
Acid	phenols	10.17918253	9.93	c1ccc(c(c1)OC)O
Acid	phenols	3.723428059	9.94	c1ccc(cc1C(=O)[O])O
Acid	phenols	9.322853815	9.95	c1(ccc(cc1)O)F
Acid	phenols	9.662906045	9.96	c1(ccc(cc1)O)O
Acid	phenols	10.21506848	9.98	c1ccc(cc1)O
Acid	phenols	10.47702901	10	c1cc(ccc1O)CC
Acid	phenols	10.53395431	10.08	c1ccc(cc1C)O
Acid	phenols	10.39534188	10.19	c1(ccc(cc1)O)C
Acid	phenols	10.13463623	10.2	c1(ccc(cc1)O)OC
Acid	phenols	10.39194137	10.2	c1cc(c(cc1)O)CC
Acid	phenols	10.42297946	10.28	c1ccc(c(c1)C)O
Acid	thiophenols	5.86741082	4.72	Sc1ccc(cc1)N(=O)=O
Acid	thiophenols	5.87091448	5.24	Sc1cc(ccc1)N(=O)=O
Acid	thiophenols	6.8153639	5.3	Sc1c[nH]c(=O)[nH]c1=O
Acid	thiophenols	5.547644575	5.33	Sc1ccc(cc1)C(=O)C
Acid	thiophenols	6.045305853	5.78	Sc1cc(ccc1)Cl
Acid	thiophenols	6.07059389	6.02	Sc1ccc(cc1)Br
Acid	thiophenols	6.06110893	6.14	Sc1ccc(cc1)Cl
Acid	thiophenols	6.56239591	6.39	c1(cc(ccc1)OC)S
Acid	thiophenols	6.353940117	6.61	Sc1ccccc1
Acid	thiophenols	6.476010874	6.64	Sc1c(cccc1)C
Acid	thiophenols	6.513214264	6.66	Sc1cc(ccc1)C
Acid	thiophenols	6.56239591	6.78	Sc1cc(ccc1)OC
Acid	thiophenols	6.488374676	6.82	Sc1ccc(cc1)C

Supplementary Table ST1: BCL-XpKaAcid predictions on acidic molecular series from Thapa and Rhagavachari. Predicted pKa values were calculated using BCL-XpKaBase retrained with 'Series' molecules removed from the training set.

Model	Series	pKa_predicted	pKa_experimental	SMILES
Base	anilines	2.550371609	0.28	c1ccc(c(c1)N(=O)=O)N
Base	anilines	3.548858506	0.98	c1(ccc(cc1)N)N(=O)=O
Base	anilines	2.676207934	2.04	c1ccc(c(c1)C(=O)O)N
Base	anilines	2.629611617	2.1	c1(ccccc1N)C(=O)OCC
Base	anilines	2.601269559	2.16	c1(ccccc1N)C(=O)OC
Base	anilines	3.399807604	2.3	c1(ccc(cc1)N)C(=O)OC
Base	anilines	3.199367608	2.32	c1(ccc(cc1)N)C(=O)O
Base	anilines	3.455025191	2.38	c1(ccc(cc1)N)C(=O)OCC
Base	anilines	3.304264101	2.45	c1ccc(cc1N(=O)=O)N
Base	anilines	3.770251455	2.62	Clc1ccccc1N
Base	anilines	3.705883575	2.96	c1ccc(c(c1)F)N
Base	anilines	3.239350563	3.05	c1ccc(cc1C(=O)O)N
Base	anilines	3.973017772	3.32	c1ccc(cc1Cl)N
Base	anilines	4.085298274	3.38	c1ccc(cc1F)N
Base	anilines	3.07294118	3.56	c1(cc(ccc1)N)C(=O)OC
Base	anilines	4.369909677	3.78	c1ccc(c(c1)c1ccccc1)N
Base	anilines	4.060990933	3.81	c1(ccc(cc1)N)Cl
Base	anilines	4.241077314	4.05	c1(cccc(c1)N)SC
Base	anilines	4.563150522	4.17	c1ccc(cc1O)N
Base	anilines	4.421831809	4.17	c1(cc(ccc1)N)OCC
Base	anilines	4.344548511	4.2	c1ccc(cc1OC)N
Base	anilines	4.600652812	4.38	c1ccc(c(c1)C)N
Base	anilines	4.389007963	4.4	c1(ccc(cc1)N)SC
Base	anilines	4.438197402	4.47	c1(c(cccc1)N)OCC
Base	anilines	4.26177323	4.49	c1ccc(c(c1)OC)N
Base	anilines	4.158660088	4.52	c1(ccc(cc1)N)F
Base	anilines	4.596235174	4.67	c1ccc(cc1C)N
Base	anilines	4.404998413	4.72	c1ccc(c(c1)O)N
Base	anilines	4.593323009	5.07	c1(ccc(cc1)N)C
Base	anilines	4.686058939	5.25	c1(ccc(cc1)N)OCC
Base	anilines	4.539499865	5.29	c1(ccc(cc1)N)OC
Base	anilines	4.500701695	5.5	c1(ccc(cc1)N)O
Base	NArHet	2.404660253	1.1	c1ccncn1
Base	NArHet	2.720903467	2.1	c1cccnn1
Base	NArHet	5.094260599	2.84	c1c(cccn1)Cl
Base	NArHet	2.527505137	3.28	c1(ccccn1)OC
Base	NArHet	2.511515911	3.39	c1cc2c(cc1)cnnc2
Base	NArHet	4.599799687	4.85	c1cc2c(cc1)cccn2
Base	NArHet	5.132039245	4.86	c1c(cccn1)O
Base	NArHet	4.517242849	4.88	c1ccc(cn1)OC
Base	NArHet	5.252557741	5.05	c1cc2c(cc1)cc1c(c2)nccc1
Base	NArHet	5.57982415	5.17	c1ccccn1
Base	NArHet	5.090379419	5.6	c1cc2c(cc1)cc1c(cccc1)n2
Base	NArHet	3.882480383	5.7	c1c(ccn1)CC
Base	NArHet	5.447206839	5.82	c1c(cccn1)C(C)(C)C

Base	NArHet	5.642966617	5.97	c1cccc(n1)CC
Base	NArHet	5.612531275	5.99	c1cc(ccn1)C(C)(C)C
Base	NArHet	5.727016144	6	c12c(cccc1)nc[nH]2
Base	NArHet	5.634219651	6.02	c1cc(ccn1)CC
Base	NArHet	6.511484139	6.45	c1[nH]cc(n1)CO
Base	NArHet	5.463276643	6.62	c1cc(ccn1)OC
Base	NArHet	6.580189592	7.05	c1(cnc[nH]1)C[C@@H](C(=O)[O])NC(=O)C
Base	NArHet	7.849208492	7.75	c1(ncc[nH]1)C
Base	1° amines	7.151574237	5.3	NCC#N
Base	1° amines	9.434636593	9.34	c1cc(ccc1)CN
Base	1° amines	10.45894373	9.68	c1cc(ccc1)CCN
Base	1° amines	10.50026728	9.8	C(N)(C)C
Base	1° amines	10.55331237	10.59	NCCCC
Base	1° amines	10.53639823	10.6	C(CN)C
Base	1° amines	10.77548936	10.63	CN
Base	1° amines	10.60940113	10.68	C(N)(C)(C)C
Base	1° amines	10.53501153	10.7	C(N)C
Base	1° amines	10.49623019	11.23	[C@@H]1(CC[C@@H](CC1)N)C(C)(C)C
Base	2° amines I	8.942531148	8.55	N(C[C@@H](c1ccc(c(c1)O)O)O)C
Base	2° amines I	10.12634812	10.54	C(NC)C
Base	2° amines I	10.08675411	10.78	CNC
Base	2° amines I	10.18338682	11	CCCNCCC
Base	2° amines I	10.15547746	11.02	C(NCC)C
Base	2° amines I	10.12571451	11.22	C1CCNCC1
Base	2° amines I	10.23048234	11.23	C(CNC1CCCCC1)C
Base	2° amines I	10.14815418	11.27	C1CCCN1
Base	2° amines II	9.310021116	9.69	C1CCN(CC1)CC=C
Base	2° amines II	9.315685373	9.8	CN(C)C
Base	2° amines II	9.356239722	10.16	C(N(C)C)C
Base	2° amines II	10.7512102	10.75	CCN(CC)CC

Supplementary Table ST2: BCL-XpKaBase predictions on basic molecular series from Thapa and Rhagavachari. Predicted pKa values were calculated using BCL-XpKaAcid retrained with 'Series' molecules removed from the training set. NArHet = Nitrogen aromatic heterocycle.
